# Global research hotspots and trends in non-surgical treatment of adolescent idiopathic scoliosis over the past three decades: a bibliometric and visualization study

**DOI:** 10.3389/fped.2023.1308889

**Published:** 2024-01-10

**Authors:** Jie Xu, Meng Chen, Xin Wang, Lin Xu, Xiaobing Luo

**Affiliations:** ^1^ Department of Sports Medicine, Sichuan Provincial Orthopedics Hospital, Chengdu, China; ^2^ Department of Emergency Medicine, Nanchong Hospital of Traditional Chinese Medicine, Nanchong, China; ^3^ Health Science Center, Peking University, Beijing, China; ^4^ Department of Outpatient Nursing, Nanchong Central Hospital, Nanchong, China

**Keywords:** adolescent idiopathic scoliosis, bibliometric analysis, brace, physiotherapy scoliosis-specific exercise, rehabilitation studies published in Psychology/Education/Social, Sports/Rehabilitation/Sport

## Abstract

**Background:**

In recent years, research on the non-surgical treatment of AIS has been increasingly conducted. To the best of our knowledge, this field doesn't yet have a comprehensive and structured pulse combing analysis. In order to provide inspiration and resources for subsequent researchers, we thus reviewed the literature studies on the non-surgical treatment of AIS from the previous thirty years and highlighted the hotspots and frontiers of research in this field.

**Methods:**

Main using Citespace 6.1 software, the data from the core dataset of the WOS database pertaining to the non-surgical management of AIS from 1990 to 2022 was gathered, displayed, and analyzed.

**Results:**

839 papers in all were included in the literature. With 215 papers, the USA came in first place. Chinese Univ Hong Kong ranked first with 32 papers. Research hotspots are adolescent idiopathic spondylitis, Schroth-based physiotherapy-specific exercise efficacy, curve development, Cobb angle, TLSO brace-based clinical efficacy, quality of life, reliability, health-related quality of life questionnaires, finite element biomechanical models, follow-up, and clinical guidelines.

**Conclusion:**

There aren't many studies that compare the clinical effectiveness of various non-surgical treatments, and because of variations in inclusion eligibility standards and outcome measures, these studies cannot be directly compared. In addition, the inconsistency of existing growth potential and progression risk assessment systems further affects comparative studies of clinical efficacy; it is recommended to establish primary assessment indicators centered on patient treatment outcomes (including appearance, disability, pain, and quality of life), as well as standardized scoliosis progression risk assessment criteria.

## Introduction

Adolescent idiopathic scoliosis (AIS) is a three-dimensional spinal deformity that develops in teenagers for unexplained causes, including serial abnormalities in the coronal, sagittal, and axial positions (1). The main diagnostic criterion is a Cobb angle of ≥10° in the coronal plane. It occurs in adolescents aged 10–17 years, with an overall prevalence of 1%–3% (2). When the Cobb angle was between 10° and 20°, the proportion of impacted girls to boys was roughly the same (1.3:1), increasing to 5.4:1 between 20° and 30° for the Cobb angle and to 7:1 for angular values above 30° (3, 4). Since adolescents are at the pinnacle of human development and growth, they are also the period when scoliosis progresses most rapidly. In addition to the effects on the spinal structure, there is also damage to the development and function of the adjacent organs of the spine, particularly the thorax and lungs. Treatment options are currently based on the treatment guidelines of the International Scientific Society on Scoliosis Orthopaedic and Rehabilitation Treatment (SOSORT) (5). Bracing is the preferred treatment option for children with Cobb's angle of 26°–45°AIS, and surgery is recommended for patients with Cobb's angles greater than 40°–45°. However, some children with AIS and their parents refuse surgery altogether and insist on wearing a brace. Recently published meta-analyses have shown that bracing can also stop the natural history of scoliosis curves from 40° to 60° (6). In recent years, a growing number of high-quality randomized controlled trials have demonstrated the positive short-term effects of Physiotherapeutic Scoliosis-specific Exercises (PSSE) in the treatment of AIS (7, 8), whereas the effectiveness of the long-term effects is unclear, and usually, PSSE is used as a support for the treatment of AIS. Adjunct to therapy. Currently, there is only one study that lists the top 100 citations for AIS in tabular form (9). In recent decades, despite the significant progress in treatment modalities and outcomes, there have been many debates, and the emergence of new theories and technologies has brought new opportunities and challenges to treatment. The use of coronal-deformity angular ratio (C-DAR), for example, eliminates the need for supine or supine lateral bending radiographs for determining the flexibility of scoliosis curves and is instrumental in planning appropriate treatment (10); FED therapy is statistically more effective than FITS therapy in improving outcomes for girls aged 11–15 years with AIS (11); automatic pressure-adjustable orthotics can improve wear quality and thus provide better biomechanical correction during the study period (12). Examining the hotspots and trends in research on non-surgical AIS treatment is crucial. As a result, this work is the first to map the scientific knowledge of research on this topic using visual research techniques by examining its hotspots, frontiers, and evolutionary trajectories, aiming to give an in-depth overview of the research status and developments in this subject, serving as a reference for researchers.

## Methods

### Search strategy and data retrieval

**Figure 1:**
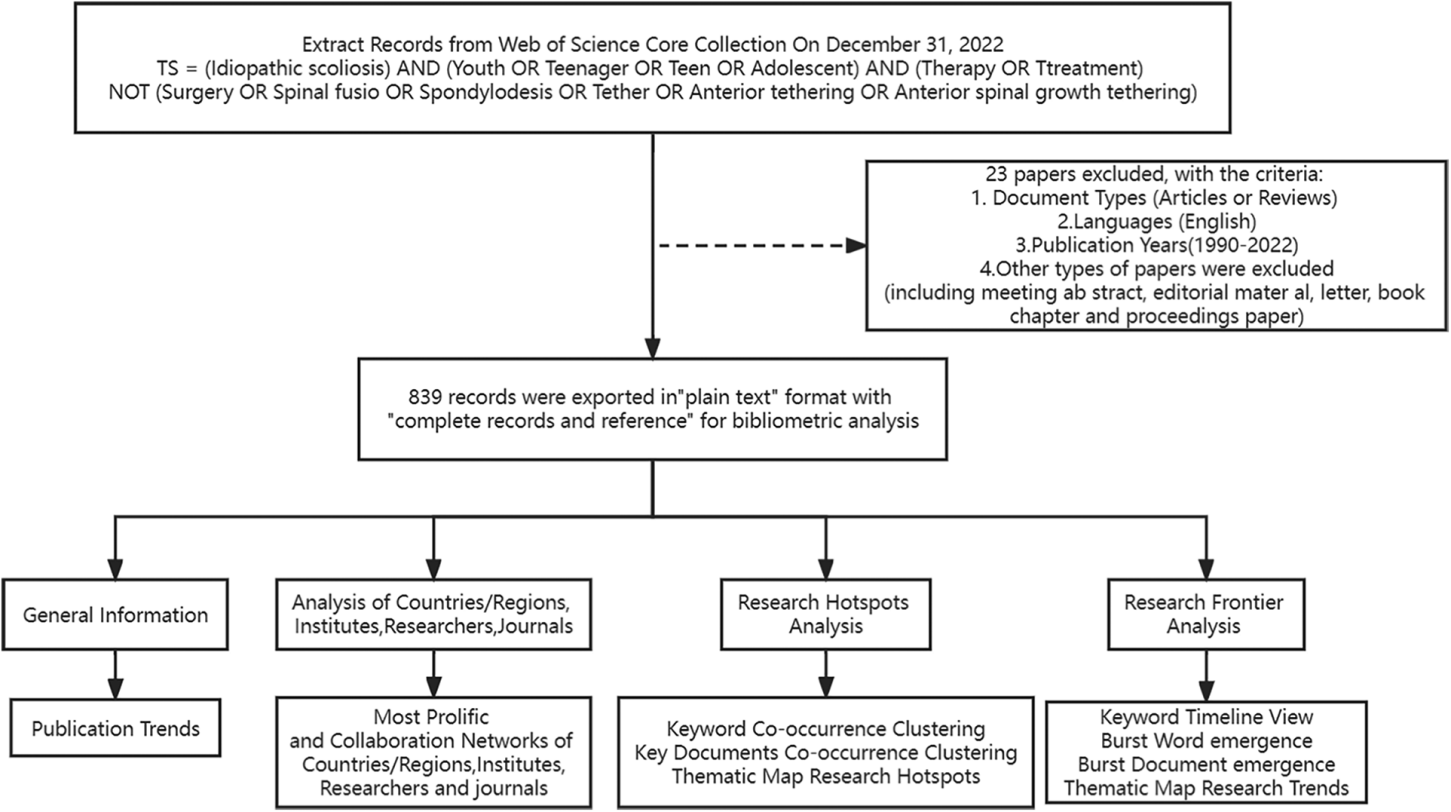
Workflow diagram.

The Web of Science database's core data set search revealed that the initial research on this topic was first published in 1980, but the number of articles from 1980 to 1990 was very small. Only ten articles were retrieved, and the research literature gradually increased from 1990, so the search time started in 1990. The search time was In the advanced search section, the search formula “TS = (Idiopathic scoliosis) AND (Youth OR Teenager OR Teen OR Adolescent) AND (Therapy OR Ttreatment) NOT (Surgery OR Spinal fusio OR Spondylodesis OR Tether OR Anterior tethering OR Anterior spinal growth tethering)” retrieved a total of 862. Inclusion criteria were: (1) the topic of the study was the non-surgical treatment of AIS; (2) the publication time of the literature was from 1990 to 01-01 to 2022-12-31; (3) the type of the literature was Article and Review, and (4) the language of the literature was English. The exclusion criteria were: (1) the topic of the study was about surgery; (2) the type of the literature was conference abstracts, news, proofreading notices, conference papers, and retraction notices; and (3) the language of the literature was not English. Twenty-three articles were excluded based on inclusion-exclusion criteria. The study encompassed 839 articles, and Figure 1 displays the process flowchart in question.

### Literature selection

The literature was read separately by two evaluators. The article's title and abstract served as the basis for the first screening, which was followed by a second screening based on the criterion for inclusion and exclusion. If there is any dispute, a third assessor reads it and decides on the final go.

### Statistical analysis methods

For the bibliometric analysis in this study, five scientometric programs—CiteSpace (6.1R6, 2023), VOSviewer (1.6.18, 2022), R-Studio based R-bibliometrix (4.6.1, 2023), Pajek (5.16, 2022), and Scimago Graphica (1.0.26, 2022)—as well as Microsoft Excel were employed. The software programs CiteSpace, created by Professor Chen C, and VOSviewer, created by Professors Van Eck and Waltman. With the use of the progressive knowledge domain visualization technique, patterns and trends in the body of scientific publication may be identified and visualized by visualizing the most referenced and important literature, areas of knowledge domain competence, and the creation of research themes (13). According to the pertinent information in the article, the data pertaining to the literature is determined using the CiteSpace visualization program (14). The counting method of VOSviewer is full count. In order to perform the function of assessing the present situation and forecasting the future, the CiteSpace emergent word detection function is utilized to investigate the keyword surge change rate and produce the table of keywords with a high rate of mutation (15). Tools like Citespace and VOSviewer are primarily employed to visualize and study the knowledge structure and development patterns of scientific research on a particular topic (16). Additionally, sub-clusters may be formed from the basic structure of the literature network by employing cluster analysis to find research sub-domains or academic hotspots (17). By using overlap analysis with the software programs R-bibliometrix, VOSviewer, and CiteSpace, it is possible to identify research frontiers that might lead to important discoveries in the next years.

Citespace visual analysis software parameters and data analysis were selected as follows to complete the collection of data through the inclusion and exclusion criteria of the literature records, and then use Web of Science to export the collected data in “plain text” format, including “complete records and references,” every 500 records to generate a file, the file is renamed in the form of “download,” and then loaded into the input folder of the Citespace program, and finally started the visualization and analysis. In the parameter settings of CiteSpace visualization software, firstly, convert the input file to the output file through the Data tab, and then select the corresponding project and data file in the project execution operation area. In the time selection area, the time span is set to 1990–2022, the time scale is set to 1 year, the filtering criterion is set to “Top N,” and the threshold is set to 50. In the Pruning option area, “Pathfinder” and “Pruning sliced networks” were selected as the clipping method to simplify the network structure and highlight important features. For node types, select country, journal, author, institution, cited author, keyword, and cited literature for co-occurrence or cluster analysis, click the “GO” option after selecting nodes, then draw a visual map, and finally fine-tuning the color scheme and font size according to the content of the map, etc. The counting method of VOSviewer is full count.

## Results

### Bibliometrics of publication output

**Figure 2:**
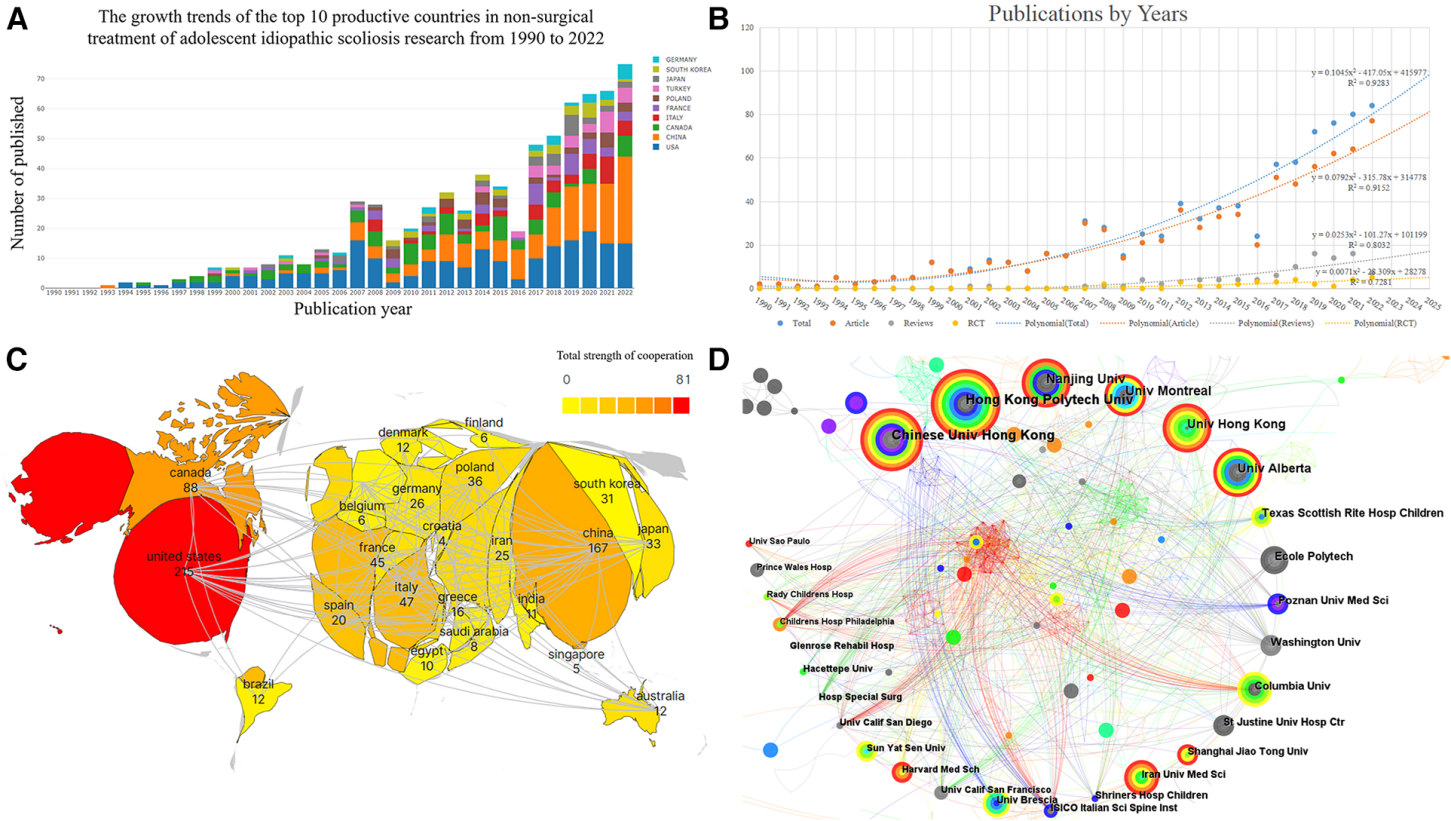
(**A**) Bibliometric analysis of the WoS core database output. The number of publications on non-surgical treatment of AIS studies published in different countries has changed year by year from 1990 to 2022. (**B**) Trends in publications in the field of non-surgical AIS research and corresponding polynomial fit curves. (**C**) Filled plot of the collaborative bibliometric analysis of countries in the field of non-surgical AIS research, with darker colors representing more publications and connecting lines representing collaborative relationships. (**D**) Institutional co-occurrence network diagram for non-surgical treatment of AIS. The circle in the chart indicates the volume of articles issued, the larger the circle indicates the more articles issued by the institution, the thickness of the purple outer circle represents the centrality of the institution, and the connecting line represents the existence of cooperation or co-occurrence.

Ultimately, 839 pertinent articles were located, and Figure 2A displays the yearly publishing output in various nations. From 1990 to 2022, the number of research articles on non-surgical management of AIS typically increased with time, with explosive growth in 2007 and 2008, when the number of publications was nearly twice that of 2006, and a trough in 2016, when the number of publications was only 60%. Overall, the US has the highest annual publication volume and is the country with the earliest start and longest duration of research in this field; China has the second highest annual publication volume, with a late start but rapid development in this field. Especially in recent years, it has increased significantly year by year and is already the country with the highest percentage of annual publications. With the help of a polynomial fit analysis, the year of publishing and the total number of articles were shown to be significantly correlated [the coefficients of determination (R2) for all papers, articles, reviews, and randomized controlled trials were 0.9283, 0.9152, 0.8032, and 0.7281, respectively]. We predict that in 2025, there will be around 98 papers published, comprising about 81 articles, 17 reviews, and 5 RCTs, as illustrated in Figure 2B, based on the polynomial fit analysis. In general, the growth of the orthopedics and rehabilitation medicine fields has prompted more study. Despite the annual increase in publications, it is evident that there are still not enough highly qualified RCT trials.

### Countries or regions and cooperation networks

The generated visualization includes a total of 53 nations or regions, of which 13 had ≥20 articles. Information about the top 10 nations in regard to the number of papers is indicated in Table 1. It is known from the figure that all three of these nations have developed strong scientific partnerships with other nations, see Figure 2C.

**Table 1:** Top 10 high-impact countries and institutions for non-surgical AIS research.

Country	Number of articles issued (articles)	Centrality	Institution	Number of articles issued	Centrality
USA	215	0.25	Chinese Univ Hong Kong	32	0.05
Peoples R China	167	0.27	Hong Kong Polytech Univ	31	0.01
Canada	88	0.15	Univ Montreal	26	0.05
Italy	47	0.05	Nanjing Univ	26	0
France	45	0.09	Univ Alberta	23	0.02
Turkey	38	0.01	Univ Hong Kong	23	0.01
Poland	36	0.08	Texas Scottish Rite Hosp Children	17	0.05
Japan	33	0	Ecole Polytech	17	0.01
South korea	31	0	Washington Univ	16	0.06
Germany	26	0.1	Poznan Univ Med Sci	16	0.03

### Research institutions and cooperation networks

1,165 institutes in all have published studies in this field, among which 11 institutions published ≥15 articles. Information on the top ten organizations in regard to the quantity of articles published is shown in Table 1. With other universities, these three institutions have developed strong research partnerships, see Figure 2D.

### High-impact authors and collaborative networks

**Figure 3:**
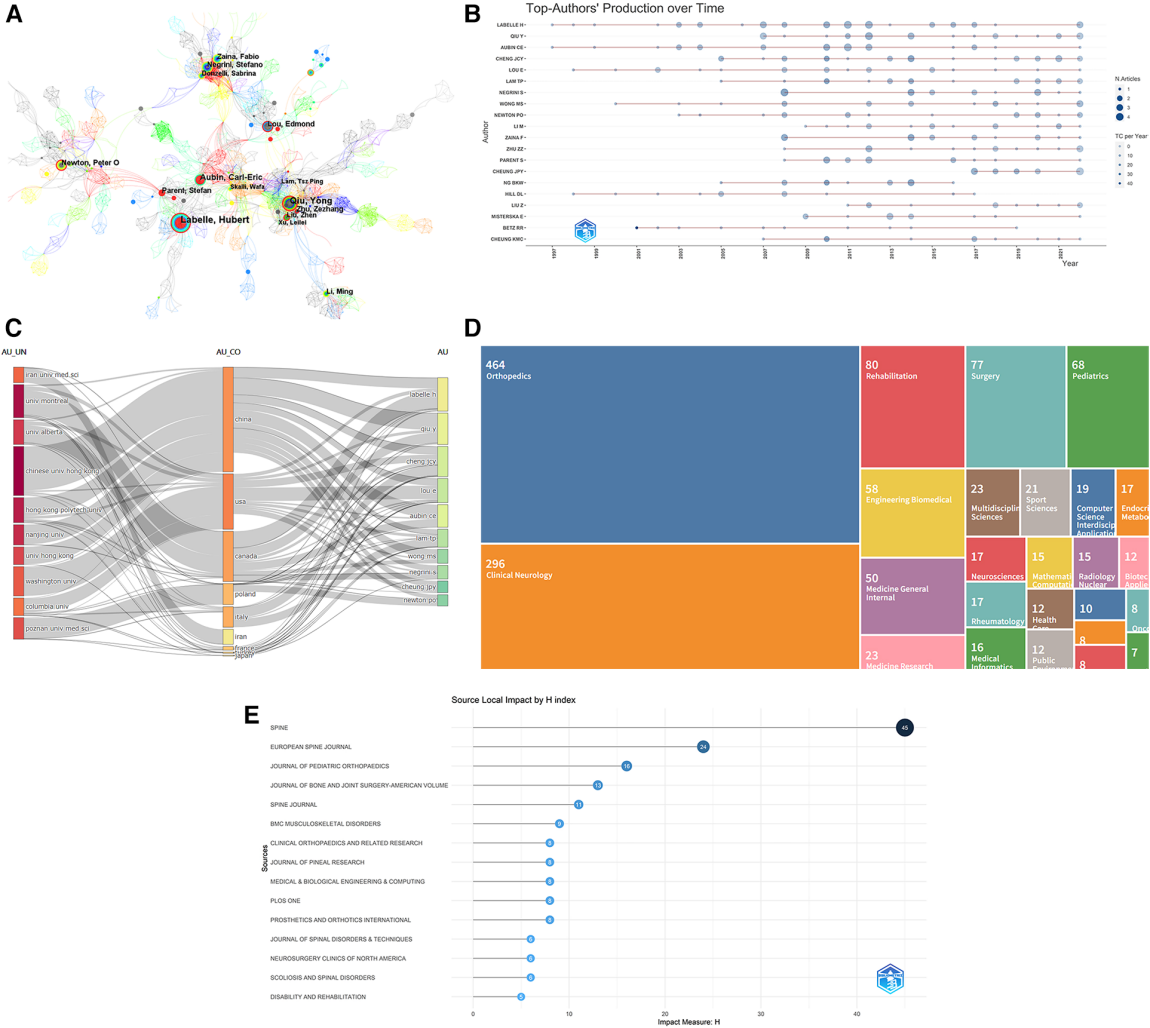
(**A**) Author co-occurrence network diagram for non-surgical treatment of AIS. The circles in the chart indicate the volume of posts, the larger the circle the more the author posts, the thickness of the purple outer circle represents the author's centrality, and the connecting line represents the existence of cooperation or co-occurrence. (**B**) Plot of high-yield authors of non-surgical treatment of AIS over time. The top twenty most prolific researchers in the field and their publications. The larger the node, the more literature is published. The darker the color, the more citations. The color represents the number of publications, and the color represents the number of citations per year. (**C**) The three-field plot showing the knowledge flow for non-surgical treatment of AIS. (**D**) Distribution of hot research disciplines in the literature of non-surgical treatment of AIS. (**E**) H-index of high-impact journals for non-surgical AIS research, 1990–2022.

Of the 3,472 authors included in the visualization atlas, 15 have published more than 10 articles. Information on the top ten scholars regarding the number of articles and the top ten scholars regarding citation frequency is displayed in Table 2. Figures 3A–C show that there is some collaboration amongst author teams, with this collaboration being more pronounced among the high-yielding writers and lacking among authors with a high centrality in the literature.

**Table 2:** Top 10 high-impact authors for non-surgical AIS studies.

Author	Country	Institution	Number of articles issued	Author	Frequency of citations	Country
Labelle, Hubert	Canada	Univ Montreal	27	Negrini S	54	Italy
Qiu, Yong	China	Gulou Hospital	24	Weinstein SL	49	USA
Aubin, Carl-Eric	Canada	Montreal Polytech Univ	16	Kuru T	22	Turkey
Lou, Edmond	Canada	Glenrose Rehabilitation Hospital	14	Berdishevsky H	20	USA
Negrini, Stefano	Italy	Scientific Spine Institute	14	Thompson RM	17	USA
Newton, Peter O	USA	San Diego Children's Hospital	14	Dunn J	17	USA
Parent, Stefan	Canada	Santo Justin Hospital	13	Negrini S	17	Italy
Zhu, Zezhang	China	Gulou Hospital	13	Nachemson AL	15	Sweden
Li, Ming	China	Changhai Hospital	12	Schreiber S	15	Canada
Zaina, Fabio	Italy	Scientific Spine Institute	12	Park JH	15	Korea

### Literature research hotspot disciplines

The distribution of research hotspots in the collected 839 documents was analyzed, and the literature was then categorized into 25 groups. From the data, it can be seen that the field has been widely researched and developed in the fields of orthopedics, clinical neurology, and rehabilitation medicine, Figure 3D.

### High-impact publications and citation patterns

**Figure 4:**
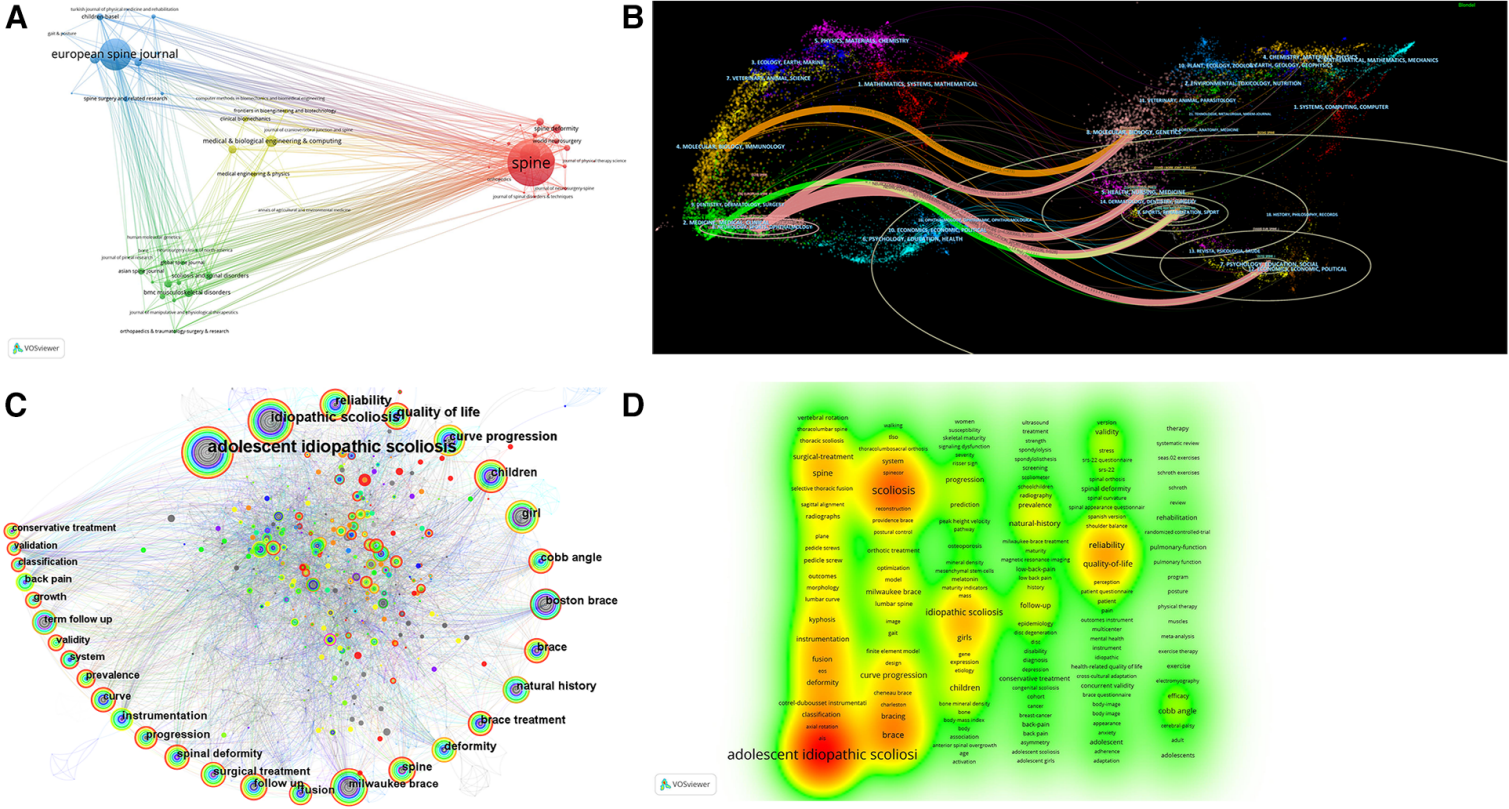
(**A**) Cluster visualization of journal co-citation analysis generated based on VOSviewer software. Each node represents a journal, and the size of each circle is determined by the journal's co-citation. (**B**) Biplot overlay of cited and cited journals in the field of non-surgical AIS research. Cited journals are on the left, cited journals are on the right, and the line represents the citation status. (**C**) Keyword co-occurrence map for non-surgical treatment of AIS. The circles in the figure represent keywords, and the larger the circle indicates the higher the frequency of the keyword. The dark to light colors represent the years from far to near, the connecting lines represent the links between the keywords, and the thickness of the purple outer circle represents the centrality of the keywords. (**D**) VOSviewer-based clustering view of non-surgical treatment AIS keywords.

**Figure 5:**
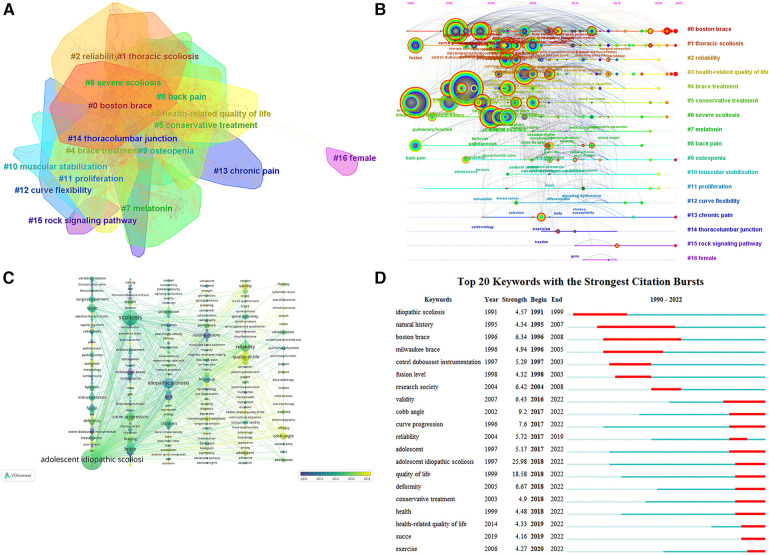
(**A**) Keyword co-occurrence clustering map for non-surgical treatment of AIS. The dark to light colors represent the years from far to near, and the connecting lines represent the links between keywords. (**B**) Keyword timeline view of non-surgical treatment of AIS. The circles in the figure represent keywords, and the larger the circle indicates the higher the frequency of keywords. The dark to light colors represent the years from far to near, the connecting lines represent the links between the keywords, and the thickness of the purple outer circle represents the centrality of the keywords. (**C**) VOSviewer-based keyword time view of non-surgical treatment AIS. (**D**) Keyword emergence map for non-surgical treatment of AIS. The figure of “▂” is a 1-year keyword mark, and “▂” is the emergence of the year of the word.

All 839 of the papers found in the search came from 262 publications. The top ten rankings of the number of publications and the ranking of the H-index are shown in Table 3, Figure 3E. According to the VOSviewer journal co-citation study, the top three citation frequencies were Spine (793 times), Journal of Bone and Joint Surgery-American Volume (587 times), and European Spine Journal (561 times), see Figure 4A. Journals that publish in this field of study currently have a significant impact. The biplot overlay's colored trails connecting journal groupings highlight the link between citing and cited journals in terms of citations, illuminating the citation trajectory and information flow (18). The colored paths indicate that studies published in Neurology/Sports/Ophthalmology journals usually cite studies published in Psychology/Education/Social, Sports/Rehabilitation/Sport, Health/Nursing/Medicine, and Molecular/Biology/Genetics. In each cluster, Figure 5 provides more details on the typical cited and cited journals. For example, the most representative journals in the Psychology/Education/Social cluster are the Spine, Arthroscopy, European Spine Journal, and Spine Journal. The most representative journals in the Sports/Rehabilitation/Sport group are the Journal of Bone and Joint Surgery-American Volume, Journal of Pediatric Orthopaedics, Clinical Orthopaedics and Related Research, and Journal of Bone and Joint Surgery-Beitish Volume.

**Table 3:** Status of high-impact journals for non-surgical AIS research, 1990–2022.

Journal name	Total number of articles	Total number of applications	Average number of citations	IF (2022)	JCR (2022)	H-index
Spine	122	816	6.69	3.241	Q1	45
European Spine Journal	77	305	3.96	2.721	Q2	24
Journal of Bone and Joint Surgery-American Volume	16	236	14.75	6.558	Q1	13
Journal of Pediatric Orthopaedics	25	124	4.96	2.537	Q2	16
Prosthetics and Orthotice International	10	88	8.8	1.672	Q3	8
Medical & Biological Engineering & Computing	10	66	6.6	3.079	Q2	8
Disability and Rehabilitation	9	51	5.67	2.439	Q1	5
Clinical Orthopaedics and Related Research	10	49	4.9	4.755	Q1	8
Plos One	11	45	4.09	3.752	Q2	8
Spine Journal	20	42	2.1	4.297	Q1	11

## Keyword visualization analysis

### Hotspot analysis of keyword co-occurrence clustering

The summary of research hotspots and the investigation of research trends depend heavily on keyword analysis (16). Figures 4C,D, Table 4 show the keywords with the highest co-occurrence. Research hotspots over the time period are indicated by terms with high centrality and frequency. According to an examination of common keywords, the primary topics of this field's research were adolescent idiopathic scoliosis, idiopathic scoliosis, quality of life, reliability, curve progression, children, girl Cobb angle, Boston brace, natural history, brace, brace treatment, deformity, spine, Milwaukee brace, follow up, etc. Figure 5A exhibits the keyword co-occurrence clustering graph in this domain. A total of 17 groups were constructed using the typical LLR algorithm, and the keyword clustering analysis indicated that the more aggregation there was, the more homogeneous the relationships between studies were (19). The cluster number and the cluster size are inversely connected, with cluster number 0 representing the largest cluster and so on. The keyword clusters from #0 to #16 were Boston brace, thoracic scoliosis, reliability, health-related quality of life, brace treatment, conservative treatment, severe scoliosis, melatonin, back pain, osteopenia, muscular stabilization, proliferation, curve flexibility, chronic pain, thoracolumbar junction Except for cluster #16, and all clusters were intertwined and closely related to each other. Keyword co-occurrence and cluster analysis yielded adolescent idiopathic spondylitis, quality of life, reliability, curve development, girls, Cobb angle, Boston brace, and brace treatment as current research hotspots in the field.

**Table 4:** Non-surgical treatment of AIS high-frequency keywords and centrality TOP10.

Keywords	Frequency	Keywords	Centrality
Adolescent idiopathic scoliosis	495	Bone mineral density	0.13
Idiopathic scoliosis	120	Children	0.08
Quality of life	108	Classification	0.08
Reliability	83	Girl	0.07
Curve progression	75	Follow up	0.07
Children	69	Prevalence	0.07
Girl	62	Prediction	0.07
Cobb angle	59	Cobb angle	0.06
Boston brace	57	Boston brace	0.06
Natural history	56	Milwaukee brace	0.06

### Analysis of research trends in keyword timeline views

A timeline representation of the literature that the WOS database has filtered allows users to see the temporal dynamics of the clustered keywords, Figures 5B,C. A strong clustering structure is shown by the clustering Modularity (*Q* value) = 0.65 > 0.5; a persuasive clustering structure is also indicated by the average cluster Silhouette (*S* value) = 0.8707 > 0.5 (20). From 1990 to 1995, the keywords nonoperative treatment, fusion, term follow up, idiopathic scoliosis, back pain, and pulmonary function received extensive attention; from 1995 to 2000, milwaukee brace, girl, follow up, boston brace, curve progression, adolescent idiopathic scoliosis, brace treatment, natural history, spine, quality of life lumbar spine, rib cage, cotrel dubousset instrumentation, growth, and adolescent keywords get attention; from 2000 to 2005 management, system, Instrumentation, spinal deformity, fixation, Cobb angle, classification, radiograph, reliability, progression, children, conservative treatment, curve, and selection keywords get attention; 2005–2010 deformity, exercise, efficiency, validation, validity, peak height velocity, prevalence, and questionnaire keywords get attention; 2010–2015 criteria, construct, interobserver reliability, health-related quality of life, spinal curvature, randomized controlled trial, risk, association, mesenchymal stem cell, school screening program, predictor, gene become new terms; 2015–2020 providence brace, succe, impact, brace questionnaire, magnetic resonance imaging, coronal balance, and expression become new terms; 2020–2022 schroth exercise, skeletally immature patient, 3D printing, mri migratio, postural balance, chondrocyte become new terms. The field is predicted to continue to delve into research around the Schroth exercise, Providence brace, skeletally immature patients, brace questionnaires, and magnetic resonance imaging.

### Analysis of research trends in emergent word emergence

The emerging words are words that are often used throughout time, showing hotspots and patterns, as seen in Figure 5D. The strongest mutation is “adolescent idiopathic scoliosis” (25.98), followed by “quality of life” (18.58), and in third place is “Cobb angle” (9.2). The keywords with the highest level of mutation during the previous three years include “health-related quality of life” (2019–2022), “succe” (2019–2022), and “exercise” (2020–2022). With the help of Burst emergence analysis and the dynamic temporal evolution of keywords, we may gain a comprehensive understanding of current and upcoming research trends in the field, where the emergent words are validity, Cobb angle, curve progression, deformity, conservative treatment, health, health-related quality of life, succe, and exercise have persisted to date and are likely to remain research hotspots.

## Visual analysis of key documents

### Analysis of research hotspots in key literature

**Figure 6:**
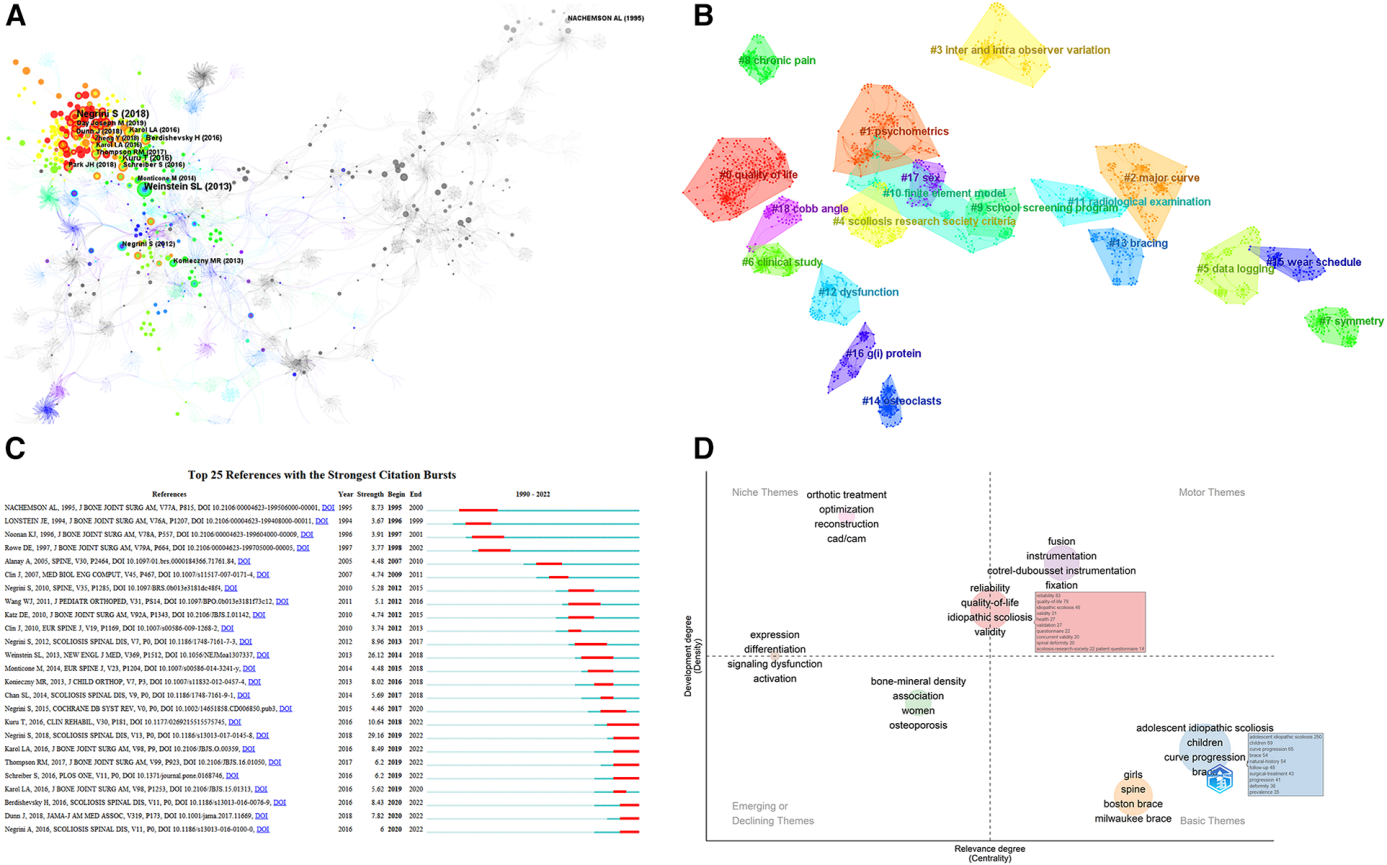
(**A**) Co-cited literature network for non-surgical treatment of AIS. The circles in the figure represent cited literature (showing the first author's name), and the larger the circle, the more citations the literature has received. The thicker the circle is, the more citations the literature has received. The line represents co-citations among the literature, and the thickness of the purple outer circle represents the centrality of the literature. (**B**) Clustering of co-cited literature for non-surgical treatment of AIS. The dark to light colors represent the years from far to near, and the connecting lines represent the links between keywords. (**C**) Emergence of key literature on non-surgical treatment of AIS. Note: The “▂” in the figure are the years in which the unexpected citations appeared, and the “▂” in the figure are the nodes in which the number of citations for the unexpected citations suddenly rose, arranged in chronological order from top to bottom. (**D**) Thematic map of non-surgical treatment AIS.

In this field, 839 documents were found, having a total citation frequency of 5,837, and the top 10 rankings of highly cited and highly centralized literature are shown in Figure 6A and Tables 5, 6. In order to identify the research hotspots and evolutionary trajectories in the field of non-surgical treatment of AIS, it is necessary to analyze the literature with the highest citation frequency and co-cite important nodes. They can be divided into three groups depending on the type of study: clinical pilot studies, clinical observational studies, and reviews. Citation frequency 2nd, 3rd, and 9th are clinical experimental studies, centrality 1st, 2nd, 4th, 5th, 6th, 9th, 10th and citation frequency 5th and 8th are clinical observational studies, and centrality 3rd, 7th, 8th and citation frequency 1st, 4th, 6th, 7th, 10th are review studies. The highly cited literature in clinical pilot studies mainly involved clinical efficacy studies with Boston (TLSO) brace as the main study (citation frequency 2nd studies) and physical therapy-specific exercise efficacy studies with Schroth as the main study (citation frequency 3rd and 9th studies). The highly cited literature in clinical observational studies is mainly about the positive effects of brace therapy (citation frequency 5th and 8th studies) and the questionnaire reliability and validity of HRQOL, mainly SRS-22, SRS-7, BSSQbrace and SAQ scales (centrality 1st, 2nd, 4th, 5th, 6th, and 10th studies). The highly cited literature in the review category was mainly research guidelines published by SOSORT (citation frequency 1st and 7th studies), clinical practice (centrality 7th study), Meta-analysis (citation frequency 10th study), and finite element analysis (centrality 8th study). For the assessment of treatment outcomes, most of the questionnaire scores of Cobb angle, angle of trunk rotation (ATR), and HRQOL were mainly used as tests. Changes in questionnaire score values for Cobb's angle, ART, and HRQOL were compared through a certain period of follow-up to illustrate the therapeutic effects of bracing and exercise interventions for AIS. By using bracing and exercise therapy for AIS patients with different curve amplitudes and skeletal maturity, the findings of every clinical study demonstrated that the use of bracing and exercise therapy had an interrupting or slowing effect on the progression of AIS, and the questionnaire score values for Cobb angle, ART, and HRQOL were significantly different from those of the observation-only control group. There are few comparative studies on the clinical efficacy of bracing and exercise therapy on AIS. The choice of treatment according to the patient's curve amplitude, skeletal maturity, and curve type, including different types of bracing, different exercise therapies, or reasonable use in conjunction with each other, should be complementary and mutually reinforcing treatments.

**Table 5:** Top 10 ranking of cited literature centrality for non-surgical treatment AIS.

Author	Centrality	Year	Title	Periodicals
Danielsson AJ (21)	0.16	2001	Health-related quality of life in patients with adolescent idiopathic scoliosis: a matched follow-up at least 20 years after treatment with brace or surgery	Eur Spine J
Bago J (22)	0.14	2004	The Spanish version of the SRS-22 patient questionnaire for idiopathic scoliosis: transcultural adaptation and reliability analysis	Spine
Altaf F (23)	0.14	2013	Adolescent idiopathic scoliosis	Bmj-Brit Med J
Caronni A (24)	0.13	2014	Improving the measurement of health-related quality of life in adolescent with idiopathic scoliosis: the SRS-7, a Rasch-developed short form of the SRS-22 questionnaire	Res Dev Disabil
Verma K (25)	0.12	2010	Demographic factors affect Scoliosis Research Society-22 performance in healthy adolescents: a comparative baseline for adolescents with idiopathic scoliosis	Spine
Botens-Helmus C (26)	0.12	2006	The reliability of the Bad Sobernheim Stress Questionnaire (BSSQbrace) in adolescents with scoliosis during brace treatment	Scoliosis Spinal Dis
Hresko MT (27)	0.09	2013	Clinical practice. Idiopathic scoliosis in adolescents	New Engl J Med
Clin J (28)	0.09	2010	Correlation between immediate in-brace correction and biomechanical effectiveness of brace treatment in adolescent idiopathic scoliosis	Spine
Bunge EM (29)	0.09	2007	Health-related quality of life in patients with adolescent idiopathic scoliosis after treatment: short-term effects after brace or surgical treatment	Eur Spine J
Carreon LY (30)	0.09	2011	Spinal appearance questionnaire: factor analysis, scoring, reliability, and validity testing	Spine

**Table 6:** Top 10 ranked frequency of cited literature for non-surgical treatment AIS.

Author	Frequency of citations	Year	Title	Periodicals
Negrini S (5)	54	2018	2016 SOSORT guidelines: orthopaedic and rehabilitation treatment of idiopathic scoliosis during growth	Scoliosis Spinal Dis
Weinstein SL (31)	49	2013	Effects of bracing in adolescents with idiopathic scoliosis	New Engl J Med
Kuru T (7)	22	2016	The efficacy of three-dimensional Schroth exercises in adolescent idiopathic scoliosis: a randomised controlled clinical trial	Clin Rehabil
Berdishevsky H (32)	20	2016	Physiotherapy scoliosis-specific exercises—a comprehensive review of seven major schools	Scoliosis Spinal Dis
Thompson RM (33)	17	2017	Brace Success Is Related to Curve Type in Patients with Adolescent Idiopathic Scoliosis	J Bone Joint Surg Am
Dunn J (34)	17	2018	Screening for Adolescent Idiopathic Scoliosis: Evidence Report and Systematic Review for the US Preventive Services Task Force	Jama-J Am Med Assoc
Negrini S (35)	17	2012	2011 SOSORT guidelines: Orthopaedic and Rehabilitation treatment of idiopathic scoliosis during growth	Scoliosis Spinal Dis
Nachemson AL (36)	15	1995	Effectiveness of treatment with a brace in girls who have adolescent idiopathic scoliosis. A prospective, controlled study based on data from the Brace Study of the Scoliosis Research Society	J Bone Joint Surg Am
Schreiber S (37)	15	2016	Schroth Physiotherapeutic Scoliosis-Specific Exercises Added to the Standard of Care Lead to Better Cobb Angle Outcomes in Adolescents with Idiopathic Scoliosis—an Assessor and Statistician Blinded Randomized Controlled Trial	Plos One
Park JH (38)	15	2018	Effects of the Schroth exercise on idiopathic scoliosis: a meta-analysis	Eur J Phys Rehab Med

Of note is a large multicenter randomized controlled trial (BrAIST) in cited frequency study 2th, which demonstrated that brace treatment was successful in stopping Cobb's angle from increasing to the surgical threshold (usually defined as ≥50°). This study has high confidence that bracing is the only strategy proven to be effective for the non-surgical treatment of AIS. In addition, in the cited frequency 5th study, the investigators compared for the first time different curve patterns between the main thoracic and main lumbar spine for the Boston thoracolumbosacral brace (TLSO) brace for AIS to investigate the efficacy of different curve patterns for the brace for AIS. Prior to the publication of this study, curve amplitude and skeletal maturity were considered to be key factors in determining the efficacy of the brace in treating AIS. However, as the study continued, it was found that even with strict adherence to the bracing protocol, between 10% and 24% of the curves still developed (39), and in patients with untreated scoliosis, the shape of the curve was linked to the risk of progression (40, 41). Therefore, this study confirms that TLSO treatment of AIS in the lumbar curve interrupts or slows the progression of scoliosis better than in the thoracic curve. Furthermore, the positive effect of Schroth physiotherapy scoliosis-specific exercise (PSSE) on slowing or interrupting the progression of AIS has been supported by randomized controlled trials (cited frequency 3rd, 9th studies), and several studies have selected different types of exercise protocols for Schroth. The Schroth best practice program is the most effective therapy for treating PSSE, according to Borysov and Mogiliantseva (42). However, in the Cited Frequency 10th study, which compared multiple Schroth exercise protocol types and exercise duration as a treatment modality for patients with Cobb's angle 10°–88° AIS, it was shown that exercise duration is a more important factor, no matter how fancy and well-designed the PSSE program. Patients should exercise for at least one month to have satisfactory clinical outcomes. Also, the Schroth exercise was more beneficial for AIS patients with Cobb angles of 10°–30° compared to AIS patients with Cobb angles greater than 30°.

Among the questionnaire studies of HRQOL, five papers selected the Scoliosis Research Society 22-item questionnaire SRS-22 (centrality 2nd, 4th, and 5th studies), the Bad Sobernheim Stress Questionnaire (BSSQbrace) (centrality 6th study), and the Spinal Appearance Questionnaire. The SRS-22 patient questionnaire has been shown to be an effective tool for the clinical assessment of patients with AIS, especially for patients who may undergo surgical interventions (43), and the widespread use of the SRS-22 in non-English-speaking countries requires its cross-cultural adaptation. The 2nd study of centrality translated and culturally adapted the SRS-22 questionnaire into Spanish, and testing demonstrated adequate internal consistency and excellent reproducibility of the Spanish version of the SRS-22 patient questionnaire. The SRS-22 questionnaire has been widely used to assess patients with scoliosis, but no studies have assessed how demographic differences in normal, unaffected adolescents affect SRS-22 scores to determine a comparative baseline for AIS. The Centrality 5th study derived baseline values for each item of the SRS-22 by assessing 450 normal adolescents. The Centrality 4th study concluded by Rasch analysis that the SRS-22 did not meet the basic measurement requirements and that the SRS-22 was affected by a severe ceiling effect when used for the first assessment of the AIS. Therefore, the SRS-7 questionnaire was modified and optimized on the basis of the SRS-22, with the advantage that it is an interval scale. BSSQbrace is a new tool to assess the stress experienced by scoliosis patients while wearing a brace, and the reliability of the BSSQbrace questionnaire was tested and validated in the centrality 6th. The Centrality 10th study described the factor analysis and scoring of the SAQ and assessed its psychometric properties.

### Analysis of research hotspots for co-occurrence clustering of key literature

On the basis of co-cited literature, cluster analysis can identify subfields that stand in for important areas of research (44). The average cluster Silhouette (*S* value) = 0.8733 > 0.5, suggesting a compelling cluster, while the clustering Modularity (*Q* value) = 0.94 > 0.5, showing a strong cluster structure (20). A total of 16 groups were constructed using the typical LLR algorithm, and the keyword clustering analysis indicated that the more aggregation there was, the more homogeneous the relationships between studies were (19). The cluster number and the cluster size are inversely connected, with cluster number 0 representing the largest cluster and so on, Figure 6B. observer variation, scoliosis research society criteria, data logging, clinical study, symmetry, chronic pain, school screening program, finite element model, radiological, examination, dysfunction, bracing, osteoclasts, wear schedule, g(i) protein, sex, Cobb angle. The field's research hotspots in terms of clustering order were exercise therapy, meta-analysis, PSSE and biomechanical models, psychometrics, major curves, interobserver differences, and Scoliosis Research Association standards; the field's research trends in terms of lighter colored clusters were quality of life, exercise therapy, meta-analysis, PSSE, biomechanical models, Cobb's angle, clinical studies, psychometrics, wear time, gender, etc.

### Analysis of research trends emerging from key literature

Burst citations are important works of literature that have been repeatedly mentioned over time, highlighting hotspots and trends, Figure 6C. The Web of Science database evaluated the 25 burst citations with the most co-cited literature, the one with the highest burst intensity was the new SOSORT 2016 guideline published by Negrini S in 2018, which focuses on idiopathic scoliosis background, descriptions of conservative treatments for different populations and flow charts for clinical practice, as well as a literature review and recommendations on assessment, bracing, PSSE and other conservative treatments (29.16); followed by Weinstein SL's 2013 randomized controlled trial and intention-to-treat trial study finding that Boston-type TLSO significantly reduced the high-risk curve progression in patients with AIS to surgical threshold, with hours of brace wear being significantly and positively associated with treatment success (26.12); in third place, Kuru T's randomized controlled trial study in 2016 found that the 3D Schroth exercise program was superior to the home exercise group and control group in the outpatient treatment group with physiotherapist supervision, particularly in terms of scoliosis angle, rotation angle, lumbar asymmetry (lumbar-elbow distance) and maximum hump height. In addition, a progression of scoliosis was observed in the control group that did not receive any treatment (10.64). According to the order in which the literature emerged, it can be broadly split into four categories: the first part focuses on studies exploring the clinical efficacy of Milwaukee braces for the treatment of AIS (1994–2002) (45–47); the second part focuses on popular theoretical studies on the pathogenesis of AIS (48) and a comparison using finite element models of the biomechanical three-dimensional effectiveness of various brace designs for the treatment of AIS (2003–2012) (49, 50); the third part of the guidelines in this field mainly describes the background, epidemiology, clinical practice process, assessment, bracing, literature review of conservative therapies such as PSSE, and recommendations (5, 35, 51) and investigate the effect of patient compliance on the clinical efficacy and HRQOL of AIS treated with braces (2013–2017) (39, 52); the fourth section is focuses on clinical efficacy studies of Schroth-based PSSE for AIS (7, 32, 37) and exploring the importance of early screening for AIS and how to efficiently detect and monitor scoliosis development during growth (2018-present) (34, 53), of which the third and fourth parts of the literature burst intensity has continued to date, and on the basis of a careful reading of the high burst citations, future research trends can be foreseen as standardization of research methods to standardize the effects of conservative treatment, the impact of patient compliance on clinical outcomes, clinical efficacy studies of PSSE, comparative efficacy studies of different Schroth types, the importance of early screening, effective modalities of early screening, the study on the success of different braces and the type of scoliosis curve, optimal use of PSSE with different braces, optimal Cobb angle for various PSSE.

### Research hotspots and trend analysis of thematic maps

The thematic maps generated based on the R-Bibliometrix package are displayed as a two-dimensional matrix. Thus, the first quadrant refers to motor topics, which are both crucial and quite well established; the second refers to niche topics, which are developed but not crucial for the domain at hand; the third refers to marginal topics, which have not been adequately established and may have just emerged and may be about to fade away; and the fourth refers to basic topics, which are crucial for the domain yet not well established, Figure 6D. Depending on which quadrant the critical bubbles are in, it can be hypothesized that fusion, orthopedic treatment, reliability, quality of life, questionnaires, effectiveness, and CAD/CAM are the hot spots for research in this area, while the themes of bone mineral density, women, osteoporosis, expression, differentiation, and signaling dysfunction may develop or vanish in the future, while in children, progression of curves, braces, girls, natural history, Boston brace, and Milwaukee brace thematic directions will require more in-depth study in the future.

## Discussion

### Bibliometric characteristics of non-surgical treatment of AIS

Bibliometrics is a scientific method first used by Alan Pritchard in 1969 (54). It helps to track data relevance and predict future boundaries. Bibliometric analysis and its visualization can effectively support the integration of information and enable researchers to understand the scope of relevant studies. In this study, a thorough bibliometric analysis of international publications on non-surgical AIS research from 1990 to 2022 was carried out. A total of 839 papers with 5,837 citations were published worldwide in the last 30 years, maintaining an overall trend of gradual increase. These results demonstrate the continued interest of researchers in the field. The two leading nations in terms of the quantity and centrality of publications on the topic are America and China, indicating that they have the greatest impact on the field. The institution with the highest number of publications is Chinese Univ Hong Kong (32), followed by Hong Kong Polytechn Univ (31). The most published scholar was Labelle, Hubert, University of Montreal, Canada (27 articles), followed by Qiu, Yong, Gulou Hospital, Nanjing University, China (24 articles). According to yearly publishing volume and trends, the US and China will keep holding the top two spots. The journal with the highest number of publications, H-index, and citations is SPINE (122 articles, 45 points, 793 citations). Data on key authors can help investigators find potential collaborators. The team represented by Labelle H, Clin J, and Phan, P, scholars from the University of Montreal, Canada, whose research areas are finite element biomechanical analysis studies of TLSO brace for AIS and studies related to the evaluation of computer-aided tools to improve brace design; the team centered on Qiu Y, Xu, L, and Cheng JC from Gulou Hospital, Nanjing University, China, whose research areas are susceptibility polymorphisms on the severity of the curve and the effectiveness of bracing in AIS patients, and the comparative study of gender differences in curve patterns, radiological characteristics, and susceptibility genes in patients with AIS; the team centered on Negrini S, Donzelli, S and Zaina, F from the Italian Scientific Spine Institute, whose areas of research were the study of the effectiveness study of the Scientific Exercise Approach to Scoliosis (SEAS) in patients with AIS at high risk of progression and the Rasch consistency study of HRQOL-related questionnaires. The team centered on Lou E, Hill DL, and Raso, James V, University of Alberta, Canada, whose members are working on the impact of patient compliance and quality of brace treatment on AIS outcomes based on a TLSO brace equipped with a wireless data collection system.

### Diversity of options and controversial efficacy of non-surgical treatment of AIS

Currently, there are two main methods of non-surgical treatment for AIS, namely, bracing and physiotherapy scoliosis-specific exercises. SOSORT recommends a non-surgical treatment schedule that selects both modalities based on Cobb angle and skeletal maturity, as shown in Table 7. A total of 14 common braces were summarized according to the characteristics of various braces for AIS, including origin, fabrication method, wearing time, curve type, mechanism of action, opening direction, symmetry, stiffness, and optimal angle, Lyon, ART (55), Boston, Rigo-Chêneau, Gensingen Brace, Milwaukee, Charleston, Providence, Wilmington, SpineCor (56), OMC (Osaka Medical College) (57), Sforzesco (58), PASB (Progressive Action Short Brace) (59), Pressure-adjustable (12), as shown in Table 8. Eight main modalities were summarized based on the main subtypes of physiotherapeutic scoliosis-specific exercises, motor intervention characteristics, classification systems, breathing techniques, and recommended brace use, Lyon (60), Schroth (61), Scientific Exercise Approach to Scoliosis (SEAS) (62), Barcelona Scoliosis Physical Therapy School (63), Dobomed (64), Side-Shift (65), Functional Individual Therapy of Scoliosis (66), FED-Method (67), as shown in Table 9. However, based on characteristics such as the volume of publications in this field of research, some trends can already be seen: In an effort to replace the most invasive braces, innovative alternative concepts have been created, with TLSO replacing Milwaukee a few years ago (68). Recently, casting has also been replaced by Sforzesco and ART braces (55). Not all of these new concepts have proven their efficacy. Efforts to gradually improve and develop some of the more established concepts continue, such as the Cheneau, Boston, or Lyon braces. However, there are also recently developed concepts, such as the OMC (57), Sforzesco (58), PASB (59), Pressure-adjustable (12), ART (55), and SpineCor (56) braces.

**Table 7:** Recommended plan for non-surgical treatment of AIS.

Adolescent	Very Low		Low		Moderate		Severe	
	Min	Max	Min	Max	Min	Max	Min	Max
Risser 0	Obs6	Obs3	Obs3	SSB	HTRB	FTRB	TTRB	Su
Risser 1	Obs6	Obs3	Obs3	SSB	PSSE	FTRB	FTRB	Su
Risser 2	Obs8	Obs6	Obs6	SSB	PSSE	FTRB	FTRB	Su
Risser 3	Obs12	Obs6	Obs6	SSB	PSSE	FTRB	FTRB	Su
Risser 4	NO	Obs12	Obs12	SIR	PSSE	FTRB	FTRB	Su
Risser 4–5	NO	Obs12	Obs12	SIR	PSSE	FTRB	FTRB	Su

Cobb degrees (Very Low: Cobb angle <10° +hump; Low: 10–20; Moderate: 21–35; Moderate to severe: 36–40; Severe: 41–50).

Obs 12/8/6/3, Observation every 12/8/6/3 months; SSB, Scoliosis soft braces; PSSE, Physiotherapeutic scoliosis-specific exercises; HTRB, Halftime rigid brace; NTRB, Night-time Rigid Bracing (8–12 h); SIR, Inpatient rehabilitation; FTRB, Full-time Rigid bracing (20–24 h) or cast; Su, Surgery.

**Table 8:** Commonly used braces for non-surgical treatment of AIS and their characteristics.

Brace	Origin (Developer)	Build method	Indicated/preferred hours of wear	Curve type	Mechanism of action	Opening	Construction envelope	Brace rigidity	Optimal indications
Lyon	France (Stagnara)	Customized	Full time	Single and double	Three point	Anterior	Symmetric	Rigid	Cobb angle ≥20° for fast growth period and ≥30° for slow growth period,11–13 years.
ART	France (de Mauroy)	Customized	Full time	Single and double	Coupled motion	Anterior	Asymmetric	Very Rigid	Cobb angle ≥ 20°
Boston	USA (Miller, Hall)	Prefabricated/Customized	Full time	Single and double	Three point	Posterior	Symmetric	Rigid	The parietal vertebra is located at T8 ∼ L2
Rigo-Chêneau	Germany (Chêneau, Rigo)	Customized	Full time	Single and double	Three point	Anterior	Asymmetric	Rigid	Cobb angle 25°–45°, Upper terminal vertebrae below T5.
Gensingen Brace	Germany (Weiss)	Customized	Full time	Single and double	Three point	Anterior	Asymmetric	Rigid	Cobb angle ≥ 40°
Milwaukee	USA (Blount)	Prefabricated/Customized	Full time	Upper thoracic, single and double	Elongation. Induced initiative	Posterior	Symmetric	Rigid	Top vertebrae at T7 and above
Charleston	USA (Reed, Cooper)	Customized	Nighttime	Single and double	Three point	Anterior	Asymmetric	Rigid	Unspecified
Providence	USA (D'Amato, McCoy)	Customized	Nighttime	Single and double	Three point	Anterior	Asymmetric	Rigid	Cobb angle ≤ 35°
Wilmington	USA (McEwen)	Customized	Full time	Single and double	Three point	Anterior	Symmetric	Rigid	Unspecified
SpineCor	Canada (Colliard, Rivard)	Prefabricated/custom fit	Full time	Single and double	Movement	dynamic strapping	Symmetric	Soft braces	Unspecified
OMC (Osaka Medical College)	Japan (Onomura)	Customized	Full time	Single and double	Three point	Posterior	Asymmetric	Rigid	Cobb angle 25–50°, and an apex of caudad to T7.
Sforzesco	Italy (Negrini, Marchini)	Customized	Full time	Single and double	three-dimensional elongation	Anterior	Symmetric	Very Rigid	From T3 to the lumbosacral region.
PASB (Progressive Action Short Brace)	Italy (Aulisa)	Customized	Full time	Only for Thoracolumbar and lumbar	Elastic deformation principle	Posterior	Symmetric	Rigid	Unspecified
Pressure-adjustable	Canada (Lou)	Customized	Full time	Single and double	Three point	Posterior	Symmetric	Rigid (Pressure adjustable)	Unspecified

**Table 9:** Main characteristics of the eight main physiotherapeutic scoliosis-specific exercises.

System Name	Main fractions	Description of the exercise intervention	Classification system	Breathing technique	Brace used
Lyon (France) (60)	1. Chaotic 2. linear	Physical therapy includes 3D activities of the spine, iliopsoas angle activities (lumbar scoliosis), patient education and activities of daily living, including sitting posture correction.	Ponseti Lenke	RAB	3D ARTbrace
Schroth (Germany) (61)	1. Thoracic scoliosis a. Thoracic spine only. b. Thorax opposite the lumbar region. c. Lumbar and hip protrusion to the other side. 2. Lumbar scoliosis a. Lumbar only with hip protrusion to the other side. b. Thoracic and hip protrusion to the other side. c. Lumbar and thoracic curve with hip in the middle. 3. Sagittal plane deformity including increased or decreased thoracic lordosis and increased lumbar lordosis or loss of anterior lordosis.	The principles followed are auto-extension (detorsion), deflection, rotation, rotary breathing and stabilization. Mirror monitoring allows the patient to synchronize corrected motion and postural perception with immediate visual feedback.	Katharina Schroth's Body Blocks	RAB	3D Chêneau brace
Scientific Exercise Approach to Scoliosis (SEAS) (Italy) (62)	1. Single curve 2. Hyperbola 3. These curve patterns are described according to the location of the top of the curve—cervicothoracic, thoracic (top above T12-L1), thoracolumbar (top at T12-L1) and lumbar (top below T12-L1), and combined double origin.	SEAS exercises are based on auto-correction and stabilization. The two main goals of the exercises are: to improve the main spinal function, i.e. spinal stability. Improvement of eventual impairments that may be emphasized by the initial assessment	Ponseti	RAB	3D Sibilla brace (Cobb <30°) Sforzesco brace (Cobb 30°−50°)
Barcelona Scoliosis Physical Therapy School(BSPTS) (Spain) (63)	1. sagittal plane deformities, such as high cervical spondylosis, inverted back and flat back. 2. structural scoliosis in the main thoracic region, which can be subdivided into: three-curve scoliosis pattern (3C), four-curve scoliosis pattern (4C), and non-3-non-4 scoliosis pattern (N3N4). 3. group 1–2 is defined as lumbar or thoracolumbar curves with a straight thoracic spine.	The correction principles follow overall postural alignment and the application of high intensity forces generated in the body, including isometric tension, dilation and specific breathing.	Katharina Schroth's Body Blocks and Manuel Rigo's radiological classification	RAB	3D Rigo Chêneau brace
Dobomed (Poland) (64)	No traditional classification system, individualized treatment plan according to the patient's condition.	The DoboMed method focuses on deepening the thoracic kyphosis, performed in a closed kinetic chain and developed on the symmetrically positioned pelvic and scapular girdle, followed by active stabilization of the corrected position and endured as a postural habit.	Dobomed	Specific Rotational angular breathing in a “phased-lock” respiration technique	3D Cheneau brace
Side-Shift (United Kingdom) (65)	Type I is any curve pattern that can be corrected by moving the trunk beyond the coronal midline to the opposite side of the scoliosis curve (a very flexible curve). 2. Type II is any curve pattern that can be corrected to the coronal midline. 3. III is any curve pattern that cannot be corrected to the midline.	The technique of the lateral shift approach is based on intensive trunk flexion training. This is an active form of auto-correction in which the patient is taught to move the trunk laterally towards the pelvis in the opposite direction to the convexity of the principal curvature	King	RAB	Milwaukee
Functional Individual Therapy of Scoliosis(FITS) (Poland) (66)	No traditional classification system, individualized treatment plan according to the patient's condition.	The FITS approach represents a functionally independent treatment for scoliosis. It mainly includes the detection and elimination of myofascial restrictions and the construction of a new series of corrective postural patterns in daily activities	NOTCS	3D corrective breathing into the concavities	3D Cheneau brace
FED-Method (Fixation, Elongation, Derotation) (Spain) (67)	There is no traditional classification system, and the focus is on young patients whose motor sensory skills have not yet fully developed and on patients with high curvature resulting in motor sensory impairment.	The FED method is described as three-dimensional stabilization of the spine with simultaneous extension and de-rotation. It is performed using a complex mechanical treatment device that allows corrective forces to act at the level of the scoliosis curve	NOTCS	RAB	FED device (passive-assisting/active-assisting)

Classification system: physical therapy with braces classification system.

NOTCS, No traditional classification system; RAB, Rotational angular breathing.

Bracing treatment affects sagittal spine pelvic parameters in adolescent AIS patients, particularly in thoracic kyphosis and lumbar lordosis (69, 70). A recent comparative study noted approximately equal rates of surgery and success between SpineCor and TLSO, with the main difference being the presence of significant advantages in health-related quality of life for the SpineCor brace, primarily in pain, self-image and functional activity subgroups of the SRS-22 questionnaire (56). A prospective randomized controlled trial with standardized follow-up according to the SRS noted a significantly higher rate of curve progression with the SpineCor brace compared to the rigid brace. Switching to a rigid brace controlled further curve progression in most patients who had previously failed with a SpineCor brace (71). The effectiveness of the SpineCor brace has not been confirmed in the literature. A systematic review found a final acceptance rate of 18% with TLSO braces, 31% with Charleston braces, and 23% with Milwaukee braces (72). Another systematic review found final acceptance rates of 12%–17% for Boston braces, 27%–41% for various braces (Boston-Charleston-TLSO), 17%–25% for nocturnal braces (Providence or Charleston braces); and 25%–33% for TLSO or Rosenberger braces; Wilmington brace 19%–30% (73). Nocturnal bracing is most effective for single lumbar/thoracolumbar curves of less than 35 degrees (74). Regardless of starting curve size and skeletal maturity, the Boston brace was found to be more effective than the Milwaukee brace by Montgomery's research (75). However, the Climent JM study concluded that Milwaukee brace-treated patients outperformed Boston braces, TLSO braces, and Charleston braces in overall Quality of Life Profile for Spine Deformities (QLPSD) scores, back flexibility, and psychosocial functioning (76). The Babaee T study found comparable differences between Milwaukee braces and thoracolumbosacral orthoses in negatively affecting the quality of life in adolescents (77). A curvilinear regression occurs with the wearing of a thoracolumbosacral orthosis and correlates with patient-reported good outcome scores (78). The traditional Lyon brace and the recently developed Sforzesco brace were contrasted in the Negrini study (58). The Sforzesco brace had better radiographic, sagittal, aesthetic, and patient recovery results than the Lyon brace. In a prospective case study, De Mauroy discovered that the ART brace produced better imaging findings than the Lyon brace, and this pattern continued after 6 months and 1 year (79). Although the ART brace demonstrated greater in-branch correction of the lumbar curve, Zaina's comparison of the short-term imaging outcomes of two superrigid braces (the Sforzesco brace and ART brace) revealed equal results (55). A two-center randomized controlled trial noted that the Pressure-adjustable brace provided better biomechanical correction over the study period by improving the quality of wear compared to the conventional brace (12). The Aulisa AG study showed that PASB-based treatment was associated with a better quality of life compared to Lyon braces (59). PASB is very effective in correcting thoracolumbar curves due to its specific biomechanical effect on vertebral body modeling (80).

However, none of these studies are directly comparable cross-sectionally due to differences in inclusion-exclusion criteria and primary endpoints used to define outcomes. It is currently unable to definitively say which brace is superior to the other, and the Scoliosis Research Society (SRS) and SOSORT are actively working to standardize AIS brace studies to address the existing controversy over the efficacy of different types of braces. Therefore, it is recommended that “patient-centered outcomes (including appearance, disability, pain, and quality of life)” be used as the primary indicator for assessing effectiveness and that uniform criteria be established for studies related to the non-surgical treatment of patients with AIS. Aiming to ensure comparability of clinical efficacy studies of different non-surgical treatments for AIS, to resolve the controversial issue of clinical efficacy of the support, and to create good conditions for later analysis of secondary reviews based on the literature base.

### Major factors affecting the efficacy of non-surgical treatment of AIS

At the time of brace initiation, the magnitude of the curve and skeletal maturity play a considerable role in predicting the success of the brace. However, as research continued, it was found that even with strict adherence to the bracing protocol, between 10% and 24% of the curves still developed (39). The risk of curve development is even greater in some patients, so the effectiveness of bracing is influenced by a variety of factors. Among the main factors are immediate correction rate, coronal deformity angular ratio, patient compliance, quality of brace treatment, initial Cobb angle, type of scoliosis, skeletal maturity, and body mass index. The factors that have a greater impact on the non-surgical treatment of AIS are the quality of brace treatment (the amount of pressure exerted by the brace) and patient compliance (the duration of brace wear).

Without controlled pressure and wear time, no meaningful conclusions about the effectiveness of the support can be drawn. Several techniques have been applied to address these issues. For example, temperature sensors (81), pressure switches (82), and force sensors (83) have been used to monitor brace pressure and/or wear time. Methods such as these have shown that the actual brace wear time is often lower than the wear time reported by the patients themselves (84). Studies with BrAIST Level I evidence suggest that brace therapy has a dose-effect relationship, i.e., as brace wear time increases, the treatment effect increases. The treatment success rate in the brace group was 90%–93%, with a mean brace duration of 12.9 h/d. The treatment success rate was 41% when the wearing time of the brace was 0–6 h per day, which was lower than the treatment success rate of the observation group (48%) (31). In addition, another prospective study also found a 100% success rate for patients wearing the brace for at least 14 h per day and a 66.7% success rate for patients wearing the brace for 2 to 10 h per day (85). Worryingly, it has been shown that brace pressure varies considerably with patient activity and posture (86). Even with increased wear time, brace pressure decreases over time (87). The pressure of the brace decreases over time, even with increased wear time (78). Therefore, even honest and compliant patients may not receive optimal results from brace therapy. The immediate rate of correction (IBC) is the most important predictor of brace efficacy, and the Xu study concluded that brace treatment is likely to fail when the IBC is below 10% (88). In contrast, a systematic review by Van de Bogaart concluded that IBC was the strongest predictor of brace success (89). The disparity between the findings of the research mentioned above may be explained by the use of various methodological standards to gauge the strength of the evidence. Coronal deformity angular ratio (C-DAR) is obtained by dividing the largest Cobb angle by the number of vertebrae involved in the same curve in the frontal plane. Lang et al. (10) used C-DAR for the first time to assess the effectiveness of wearing a 6-week short-term Gensingen brace in treating patients with AIS and found that with an initial C-DAR value of <5 and an IBC rate of >50%, the brace therapy may be successful. However, if the value is >6 and the IBC rate is <50%, the effectiveness of the brace will be reduced. Babaee T et al. (90) used C-DAR to assess the outcome of patients with AIS who wore a 2-year long-term Milwaukee brace and found that 63.9%, 29.2%, and 16.9% of patients had an IBC ≥ 50% at C-DAR <5, 5–6, and >6, respectively, and that the success rate of the brace was 89.2% for patients with an IBC ≥ 50%. That is, C-DAR can be used as a predictor of short- and long-term outcomes of AIS brace therapy. Although the initial Cobb angle and growth stage play a major role in the advancement of scoliosis in the natural disease history, it's not apparent if the initial Cobb angle is associated with the success of brace treatment. One of the three main risk factors for brace treatment failure, according to some academics, is an initial Cobb angle greater than 30° (91). The initial Cobb angle, however, has been linked to treatment success rather than failure, according to a systematic study (89). A systematic review has shown that the type of scoliosis can be a predictor of the success of brace therapy (88). The Thompson study showed that a higher proportion of patients with primary thoracic curvature progressed to a Cobb angle of ≥50° with similar initial Cobb angles and daily brace wear times (92). In contrast, some studies have also not identified the type of scoliosis as a significant factor affecting prognosis (93). The most commonly used skeletal maturity parameter is the Risser rating, and studies have found lower Risser scores in patients who failed brace therapy (88). The Karol study's findings revealed that patients with a Cobb angle >30° had a 63.0% and 32.4% risk of surgery for Risser grade 0 and open and closed acetabular “Y” cartilage, respectively (94). The Hawary study identified low skeletal maturity as a risk factor for failure of brace treatment (91). Body mass index (BMI) is an important indicator to evaluate the nutritional status of the body. A review of studies suggests that there is limited evidence supporting the association of low BMI with orthotic failure, while the evidence supporting the association of high BMI with orthotic failure is controversial (89). The Karol study found that low-weight patients wore the brace for the longest time per day (up to 15.7 h/d) and had better compliance, but they had the highest risk of surgery (60% surgery rate); overweight and obese patients wore the brace for less time per day (11.7 h/d and 9.0 h/d, respectively), while their surgery rates were 28.6% and 55.6%, respectively (95). The efficacy of bracing in the treatment of AIS is definite and has been widely used in the clinic, but the limitations of bracing treatment need to be recognized. Clinical treatment should focus on improving patient compliance, reducing complications, and providing psychological support to patients.

### Diversity of AIS progress risk assessment methods

With strict nonoperative treatment, some patients still end up having to undergo surgery for scoliosis progression, partly due to inaccurate assessment of the risk of progression in AIS patients during the development of nonoperative treatment plans (96). Therefore, the prognosis of AIS patients should be improved by developing different treatment strategies based on an accurate assessment of the risk of scoliosis progression in clinical care. There are numerous methods for assessing growth potential, each with its own strengths and weaknesses. In children who have been diagnosed, assessment of growth potential is essential to determine the stage and approach to treatment. The assessment of growth potential and risk of scoliosis progression in AIS patients reported in the literature is multidimensional and includes mainly skeletal system assessment and anthropometric assessment.

Skeletal system assessment indexes are clinically important in predicting growth potential and scoliosis progression in AIS patients. The commonly used skeletal system assessment indexes in clinical practice include Y-triangle cartilage, Risser's sign, Tanner-Whitehouse III scoring system, digital skeletal age (DSA) scoring system, distal radius and ulnar (DRU) scoring system, The degree of ossification of the Y-triangle cartilage is an important parameter for early prediction of the risk of scoliosis progression in AIS patients. The ossification of the Y-triangle cartilage mostly begins at Risser's sign level 0 and is, therefore, more closely related to the peak height velocity (PHV). It was found that the Y-triangle cartilage was open or incompletely closed in most patients with AIS at the time of PHV, suggesting that open Y-triangle cartilage predicts that the patient is still located before PHV and has a higher risk of scoliosis progression (97). The Risser's sign assesses the degree of ossification of the iliac crest epiphysis and is the most commonly used indicator of bony maturity in the assessment of the risk of scoliosis progression in AIS patients. The lower the Risser's sign, the higher the growth potential and the higher the risk of scoliosis progression. Nault proposed a modified Risser's sign classification, dividing Risser's sign grade 0 into two groups according to the closure of Y cartilage or not, and found a good correlation between Risser's sign grade 0 with Y triangle cartilage closure and Risser's sign grade 1 and DSA score 400–425 (98). This stage is when the iliac crest is just starting to ossify and is, therefore, closely related to the period of rapid scoliosis progression. However, it has been reported in the literature that approximately 40% of patients with AIS have varying degrees of abnormal iliac crest ossification, which, together with the lack of a uniform international standard for grading abnormal ossification, seriously affects the determination of Risser's sign grade. More importantly, there are currently two scoring standards for Risser's sign in this field, American and European, both of which use a 6-point scale and thus are highly confusing in the literature. Among the assessment indexes of the skeletal system, except for the Y-triangle cartilage and Risser's sign, which are simple and convenient to apply, all other assessment indexes are more complex and difficult to master in a short time. For a brief introduction to other assessment metrics, the Tanner-Whitehouse III scoring system was proposed by Tanner and Whitehouse in 1976, and its scoring criteria are based on the different morphologies of the ulnar, radial, metacarpal, and terminal phalangeal epiphyses (99). In 2007, Sanders found that the ulnar and radial bone age scores in the Tanner-Whitehouse III scoring system had the lowest correlation with growth potential, so the bone age scores of the ulna and radius in this scoring system were removed. Removed and only metaphyseal and phalangeal morphologic scores were calculated and redefined as the DSA scoring system (100). More recently, Verma further refined the DSA grading into 8 levels and defined it as the SSMS (simplified skeletal maturity scoring) scoring system (101). Hung proposed the thumb ossification composite index (TOCI) in 2017 based on the DSA scoring, which is simpler and faster and can greatly improve clinical efficiency (102). Among them, the TOCI5 level suggests that most adolescents are at the peak of height growth rate (70.1%–71.8%) (103). In contrast to the DSA score, Luk redefined and reclassified the morphology of the distal radial and ulnar epiphyses in 2014 and developed a new DRU scoring system (104). They found that the growth spurt in patients with AIS occurred at distal radial epiphysis score R7 and distal ulnar epiphysis score U5 and stopped at R10 and U9, respectively. The Greulich-Pyle mapping method is a standardized mapping of bone age based primarily on the appearance and disappearance of wrist ossification centers with age and the appearance and disappearance of the diaphysis (100). The Sauvegrain bone age score is based on morphological changes in the epiphysis of the elbow at four sites: the epicondyle, the humeral glide, the ulnar eminence, and the proximal radius on frontal and lateral radiographs of the elbow (105). The Olecranon method is a morphologic grading method based on the degree of ossification of the ulnar eminence epiphysis (106).

Anthropometric indicators commonly used to assess the risk of scoliosis progression in AIS patients include age at live birth, age at menarche, longitudinal growth rate, response to initial brace treatment, and secondary sex characteristics. Anthropometric indicators are easy to obtain, easy to visualize, and do not require additional radiation exposure, making them indispensable for assessing the risk of scoliosis progression in AIS patients. The most commonly used and simplest indicator for assessing the risk of scoliosis progression in AIS patients is the age at which Abbassi concluded that the onset of rapid height growth occurs at the age of 9 and 11 years for females and males, respectively, and that PHV occurs around 11.5 and 13.5 years for females and males, respectively. Solid footage between 11 and 13 years predicts a high risk for the development of PHV and the progression of scoliosis (107). Mao found that a significantly higher proportion of AIS patients had menarche later than 14 years of age, that menarche occurred on average 7 months (6 months to 2 years) after PHV, and that the onset of menarche represents a deceleration of longitudinal height growth (108). The risk of scoliosis progression was highest in AIS patients between 2 years before and at menarche. The longitudinal growth rate is divided into the longitudinal growth rate of height and the longitudinal growth rate of sitting height. The average PHV reported in the literature is about 8–9 cm/year, and the duration of PHV is about 3–4 years, so AIS patients about 2 years before PHV and 1.5 years after PHV have a higher risk of scoliosis progression (109). Secondary sexual characteristics are most commonly used as a visual indicator to assess the developmental maturity of AIS patients in clinical practice and are most commonly graded by the Tanner sign. When PHV occurs, male patients have a Tanner sign of approximately grade 3–5, whereas female patients tend to have a Tanner grading of grade 2–3 (99). In summary, the risk of scoliosis progression in AIS patients is closely related to their growth potential, and it is important to develop new models that include multiple dimensions with higher predictive sensitivity in the assessment.

## Limitations of the study

Only the English language literature study from the WOS database's core dataset was used in this investigation. As a result, it might have overlooked excellent literature on this topic in other databases or other languages. In the process of developing visualization atlases, there is currently no standard setup procedure for time splitting, thresholds, and cropping approaches, which could result in bias.

## Conclusion

This study offers fresh views for a rapid understanding of the area of non-surgical treatment of AIS by providing the first bibliometric and visual analysis of non-surgical treatment of AIS research during the previous 20 years from several viewpoints. Keywords of research Hotspots were adolescent idiopathic spondylitis, Schroth-based physiotherapy-specific exercise efficacy, curve development, Cobb angle, TLSO brace-based clinical efficacy, quality of life, reliability, questionnaires for HRQOL, finite element biomechanical models, follow-up and clinical guidelines. Keywords of research trends were Schroth exercise, PSSE comparative clinical efficacy studies, standardization of research methods for conservative treatment outcomes, psychometrics, the impact of patient compliance on clinical outcomes, the importance of early screening, wear time, gender, biomechanical modeling, and meta-analysis. Currently, there are fewer studies comparing the clinical efficacy of different non-surgical therapies, and the vast majority of them are not directly comparable due to differences in eligibility criteria and primary endpoints used to define outcomes, and it is recommended that “patient-centered outcomes (including appearance, disability, pain, and quality of life)” be used as the primary indicator to assess effectiveness, and that uniform criteria be established for studies related to the non-surgical treatment of AIS. Early intervention at the peak of growth is important for the prevention and treatment of AIS, but the inconsistency of existing growth potential and progression risk assessment systems may also affect comparative studies of clinical outcomes and even miss the optimal stage of intervention as a result. Therefore, it is necessary to standardize the criteria for assessing the risk of scoliosis progression. The efficacy of non-surgical treatment of AIS is influenced by multiple factors, so the clinical efficacy of different treatments cannot be generalized, and the causes should be analyzed from multiple perspectives.

## Data Availability

The raw data supporting the conclusions of this article will be made available by the authors, without undue reservation.

## References

[1] Byrd JR Scoles PV Winter RB Bradford DS Lonstein JE Moe JH. Adult idiopathic scoliosis treated by anterior and posterior spinal fusion. J Bone Joint Surg Am. (1987) 69:843–50. 10.2106/00004623-198769060-00008 3597497

[2] Zheng Y Dang Y Wu X Yang Y Reinhardt JD He C Epidemiological study of adolescent idiopathic scoliosis in eastern China. J Rehabil Med. (2017) 49:512–9. 10.2340/16501977-2240 28537345

[3] Parent S Newton PO Wenger DR. Adolescent idiopathic scoliosis: etiology, anatomy, natural history, and bracing. Instr Course Lect. (2005) 54:529–36. 15948477

[4] Lonstein JE. Scoliosis: surgical versus nonsurgical treatment. Clin Orthop Relat Res. (2006) 443:248–59. 10.1097/01.blo.0000198725.54891.73 16462448

[5] Negrini S Donzelli S Aulisa AG Czaprowski D Schreiber S de Mauroy JC 2016 SOSORT guidelines: orthopaedic and rehabilitation treatment of idiopathic scoliosis during growth. Scoliosis Spinal Disord. (2018) 13:3. 10.1186/s13013-017-0145-8 29435499 PMC5795289

[6] Babaee T Moradi V Hashemi H Shariat A Anastasio AT Khosravi M Does bracing control the progression of adolescent idiopathic scoliosis in curves higher than 40°? A systematic review and meta-analysis. Asian Spine J. (2023) 17:203–12. 10.31616/asj.2022.0162 36382367 PMC9977970

[7] Kuru T Yeldan İ Dereli EE Özdinçler AR Dikici F Çolak İ. The efficacy of three-dimensional Schroth exercises in adolescent idiopathic scoliosis: a randomised controlled clinical trial. Clin Rehabil. (2016) 30:181–90. 10.1177/0269215515575745 25780260

[8] Zaina F Negrini S Atanasio S Fusco C Romano M Negrini A. Specific exercises performed in the period of brace weaning can avoid loss of correction in Adolescent Idiopathic Scoliosis (AIS) patients: winner of SOSORT's 2008 award for best clinical paper. Scoliosis. (2009) 4:8. 10.1186/1748-7161-4-8 19351395 PMC2672077

[9] Zhao T Li Y Dai Z Zhang J Zhang L Shao H Bibliometric analysis of the scientific literature on adolescent idiopathic scoliosis. World Neurosurg. (2021) 151:e265–77. 10.1016/j.wneu.2021.04.020 33872841

[10] Lang C Huang Z Zou Q Sui W Deng Y Yang J. Coronal deformity angular ratio may serve as a valuable parameter to predict in-brace correction in patients with adolescent idiopathic scoliosis. Spine J. (2019) 19:1041–7. 10.1016/j.spinee.2018.12.002 30529785

[11] Trzcińska S Koszela K Kuszewski M. Effectiveness of the fed method in the treatment of idiopathic scoliosis of girls aged 11–15 years. Int J Environ Res Public Health. (2021) 19:65. 10.3390/ijerph19010065 PMC8750974 35010330

[12] Lin Y Lou E Lam TP Cheng JC Sin SW Kwok WK The intelligent automated pressure-adjustable orthosis for patients with adolescent idiopathic scoliosis: a bi-center randomized controlled trial. Spine (Phila Pa 1976). (2020) 45:1395–402. 10.1097/BRS.0000000000003559 32453223

[13] Chen C Song M. Visualizing a field of research: a methodology of systematic scientometric reviews. Plos One. (2019) 14:e223994. 10.1371/journal.pone.0223994 PMC6822756 31671124

[14] Xu J Du W Xue X Chen M Zhou W Luo X. Global research trends on platelet-rich plasma for tendon and ligament injuries from the past two decades: a bibliometric and visualized study. Front Surg. (2023) 10:1113491. 10.3389/fsurg.2023.1113491 PMC9950278 36843990

[15] Synnestvedt MB Chen C Holmes JH. Citespace II: visualization and knowledge discovery in bibliographic databases. AMIA Annu Symp Proc. (2005) 2005:724–8. 16779135 PMC1560567

[16] Chen C Hu Z Liu S Tseng H. Emerging trends in regenerative medicine: a scientometric analysis in citespace. Expert Opin Biol Ther. (2012) 12:593–608. 10.1517/14712598.2012.674507 22443895

[17] Chen C. Science mapping: a systematic review of the literature. J Data Inf Sci. (2017) 2:1–40. 10.1515/jdis-2017-0006

[18] Chen C Leydesdorff L. Patterns of connections and movements in dual-map overlays: a new method of publication portfolio analysis. J Assoc Inf Sci Technol. (2014) 65:334–51. 10.1002/asi.22968

[19] He J He L Geng B Xia Y. Bibliometric analysis of the top-cited articles on unicompartmental knee arthroplasty. J Arthroplasty. (2021) 36:1810–8. 10.1016/j.arth.2020.11.038 33423879

[20] Xia D Yao R Wang S Chen G Wang Y. Mapping trends and hotspots regarding clinical research on COVID-19: a bibliometric analysis of global research. Front Public Health. (2021) 9:713487. 10.3389/fpubh.2021.713487 34497794 PMC8419357

[21] Danielsson AJ Wiklund I Pehrsson K Nachemson AL. Health-related quality of life in patients with adolescent idiopathic scoliosis: a matched follow-up at least 20 years after treatment with brace or surgery. Eur Spine J. (2001) 10:278–88. 10.1007/s005860100309 11563612 PMC3611508

[22] Bago J Climent JM Ey A Perez-Grueso FJ Izquierdo E. The Spanish version of the SRS-22 patient questionnaire for idiopathic scoliosis: transcultural adaptation and reliability analysis. Spine (Phila Pa 1976). (2004) 29:1676–80. 10.1097/01.brs.0000132306.53942.10 15284516

[23] Altaf F Gibson A Dannawi Z Noordeen H. Adolescent idiopathic scoliosis. Br Med J. (2013) 346:f2508. 10.1136/bmj.f2508 23633006

[24] Caronni A Zaina F Negrini S. Improving the measurement of health-related quality of life in adolescent with idiopathic scoliosis: the SRS-7, a rasch-developed short form of the SRS-22 questionnaire. Res Dev Disabil. (2014) 35:784–99. 10.1016/j.ridd.2014.01.020 24521663

[25] Verma K Lonner B Hoashi JS Lafage V Dean L Engel I Demographic factors affect scoliosis research society-22 performance in healthy adolescents: a comparative baseline for adolescents with idiopathic scoliosis. Spine (Phila Pa 1976). (2010) 35:2134–9. 10.1097/BRS.0b013e3181cb474f 20508549

[26] Botens-Helmus C Klein R Stephan C. The reliability of the Bad Sobernheim Stress Questionnaire (BSSQbrace) in adolescents with scoliosis during brace treatment. Scoliosis. (2006) 1:22. 10.1186/1748-7161-1-22 17176483 PMC1764899

[27] Hresko MT. Clinical practice. Idiopathic scoliosis in adolescents. N Engl J Med. (2013) 368:834–41. 10.1056/NEJMcp1209063 23445094

[28] Clin J Aubin C Sangole A Labelle H Parent S. Correlation between immediate in-brace correction and biomechanical effectiveness of brace treatment in adolescent idiopathic scoliosis. Spine (Philadelphia, Pa. 1976). (2010) 35:1706–13. 10.1097/BRS.0b013e3181cb46f6 21330954

[29] Bunge EM Juttmann RE de Kleuver M van Biezen FC de Koning HJ. Health-related quality of life in patients with adolescent idiopathic scoliosis after treatment: short-term effects after brace or surgical treatment. Eur Spine J. (2007) 16:83–9. 10.1007/s00586-006-0097-9 16609857 PMC2198892

[30] Carreon LY Sanders JO Polly DW Sucato DJ Parent S Roy-Beaudry M Spinal appearance questionnaire. Spine (Phila Pa 1976). (2011) 36:E1240–4. 10.1097/BRS.0b013e318204f987 21343853

[31] Weinstein SL Dolan LA Wright JG Dobbs MB. Effects of bracing in adolescents with idiopathic scoliosis. N Engl J Med. (2013) 369:1512–21. 10.1056/NEJMoa1307337 24047455 PMC3913566

[32] Berdishevsky H Lebel VA Bettany-Saltikov J Rigo M Lebel A Hennes A Physiotherapy scoliosis-specific exercises—a comprehensive review of seven major schools. Scoliosis Spinal Disord. (2016) 11:20. 10.1186/s13013-016-0076-9 PMC4973373 27525315

[33] Thompson RM Hubbard EW Jo C Virostek D Karol LA. Brace success is related to curve type in patients with adolescent idiopathic scoliosis. J Bone Joint Surg Am. (2017) 99:923–8. 10.2106/JBJS.16.01050 28590377

[34] Dunn J Henrikson NB Morrison CC Blasi PR Nguyen M Lin JS. Screening for adolescent idiopathic scoliosis. JAMA. (2018) 319:173. 10.1001/jama.2017.11669 29318283 PMC13480065

[35] Negrini S Aulisa AG Aulisa L Circo AB de Mauroy JC Durmala J 2011 SOSORT guidelines: orthopaedic and rehabilitation treatment of idiopathic scoliosis during growth. Scoliosis. (2012) 7:3. 10.1186/1748-7161-7-3 22264320 PMC3292965

[36] Nachemson AL Peterson LE. Effectiveness of treatment with a brace in girls who have adolescent idiopathic scoliosis. A prospective, controlled study based on data from the brace study of the scoliosis research society. J Bone Joint Surg Am. (1995) 77:815–22. 10.2106/00004623-199506000-00001 7782353

[37] Schreiber S Parent EC Khodayari Moez E Hedden DM Hill DL Moreau M Schroth physiotherapeutic scoliosis-specific exercises added to the standard of care lead to better cobb angle outcomes in adolescents with idiopathic scoliosis—an assessor and statistician blinded randomized controlled trial. Plos One. (2016) 11:e168746. 10.1371/journal.pone.0168746 PMC5198985 28033399

[38] Park J Jeon H Park H. Effects of the Schroth exercise on idiopathic scoliosis: a meta-analysis. Eur J Phys Rehabil Med. (2018) 54:440–9. 10.23736/S1973-9087.17.04461-6 28976171

[39] Karol LA Virostek D Felton K Wheeler L. Effect of compliance counseling on brace use and success in patients with adolescent idiopathic scoliosis. J Bone Joint Surg Am. (2016) 98:9–14. 10.2106/JBJS.O.00359 26738898

[40] Weinstein SL Zavala DC Ponseti IV. Idiopathic scoliosis: long-term follow-up and prognosis in untreated patients. J Bone Joint Surg Am. (1981) 63:702–12. 10.2106/00004623-198163050-00003 6453874

[41] Weinstein SL Dolan LA Spratt KF Peterson KK Spoonamore MJ Ponseti IV. Health and function of patients with untreated idiopathic scoliosis: a 50-year natural history study. JAMA. (2003) 289:559–67. 10.1001/jama.289.5.559 12578488

[42] Borysov M Mogiliantseva T. Rehabilitation of adolescents with scoliosis during growth—preliminary results using a novel standardized approach in Russia. (Methodology). Curr Pediatr Rev. (2016) 12:31–5. 10.2174/1573396312666151117120746 26573163

[43] Babaee T Moradi V Shariat A Anastasio AT Khani A Bagheri M Disease-specific outcome measures evaluating the health-related quality of life of children and adolescents with idiopathic scoliosis and Scheuermann’s kyphosis: a literature review. Spine Surg Relat Res. (2022) 6:197–223. 10.22603/ssrr.2021-0237 35800626 PMC9200414

[44] Rodriguez A Laio A. Machine learning. Clustering by fast search and find of density peaks. Science. (2014) 344:1492–6. 10.1126/science.1242072 24970081

[45] Lonstein JE Winter RB. The Milwaukee brace for the treatment of adolescent idiopathic scoliosis. A review of one thousand and twenty patients. J Bone Joint Surg Am. (1994) 76:1207–21. 10.2106/00004623-199408000-00011 8056801

[46] Noonan KJ Weinstein SL Jacobson WC Dolan LA. Use of the Milwaukee brace for progressive idiopathic scoliosis. J Bone Joint Surg Am. (1996) 78:557–67. 10.2106/00004623-199604000-00009 8609134

[47] Rowe DE Bernstein SM Riddick MF Adler F Emans JB Gardner-Bonneau D. A meta-analysis of the efficacy of non-operative treatments for idiopathic scoliosis. J Bone Joint Surg Am. (1997) 79:664–74. 10.2106/00004623-199705000-00005 9160938

[48] Wang WJ Yeung HY Chu WC Tang NL Lee KM Qiu Y Top theories for the etiopathogenesis of adolescent idiopathic scoliosis. J Pediatr Orthop. (2011) 31:S14–27. 10.1097/BPO.0b013e3181f73c12 21173615

[49] Clin J Aubin CE Labelle H. Virtual prototyping of a brace design for the correction of scoliotic deformities. Med Biol Eng Comput. (2007) 45:467–73. 10.1007/s11517-007-0171-4 17370098

[50] Clin J Aubin CE Parent S Sangole A Labelle H. Comparison of the biomechanical 3d efficiency of different brace designs for the treatment of scoliosis using a finite element model. Eur Spine J. (2010) 19:1169–78. 10.1007/s00586-009-1268-2 20094736 PMC2900013

[51] Konieczny MR Senyurt H Krauspe R. Epidemiology of adolescent idiopathic scoliosis. J Child Orthop. (2013) 7:3–9. 10.1007/s11832-012-0457-4 24432052 PMC3566258

[52] Chan SL Cheung KM Luk KD Wong KW Wong MS. A correlation study between in-brace correction, compliance to spinal orthosis and health-related quality of life of patients with adolescent idiopathic scoliosis. Scoliosis. (2014) 9:1. 10.1186/1748-7161-9-1 24559234 PMC3996075

[53] Negrini A Donzelli S Maserati L Zaina F Villafañe JH Negrini S. Junctional kyphosis: how can we detect and monitor it during growth? Scoliosis Spinal Disord. (2016) 11:38. 10.1186/s13013-016-0100-0 27785477 PMC5073409

[54] Wang Y Li W Wu H Han Y Wu H Lin Z Global status and trends in gout research from 2012 to 2021: a bibliometric and visual analysis. Clin Rheumatol. (2023) 42:1371–88. 10.1007/s10067-023-06508-9 36662336 PMC9852810

[55] Zaina F de Mauroy JC Donzelli S Negrini S. SOSORT award winner 2015: a multicentre study comparing the sport and art braces effectiveness according to the SOSORT-SRS recommendations. Scoliosis. (2015) 10:23. 10.1186/s13013-015-0049-4 26265932 PMC4532257

[56] Ersen O Bilgic S Koca K Ege T Oguz E Bilekli AB. Difference between Spinecor brace and Thoracolumbosacral orthosis for deformity correction and quality of life in adolescent idiopathic scoliosis. Acta Orthop Belg. (2016) 82:710–4. 29182110

[57] Kuroki H Inomata N Hamanaka H Higa K Chosa E Tajima N. Efficacy of the Osaka Medical College (OMC) brace in the treatment of adolescent idiopathic scoliosis following scoliosis research society brace studies criteria. Scoliosis. (2015) 10:12. 10.1186/s13013-015-0036-9 25932040 PMC4415349

[58] Negrini S Marchini G. Efficacy of the symmetric, patient-oriented, rigid, three-dimensional, active (SPoRT) concept of bracing for scoliosis: a prospective study of the Sforzesco versus Lyon brace. Eura Medicophys. (2007) 43:171–81. 183–4. 16955065

[59] Aulisa AG Guzzanti V Perisano C Marzetti E Specchia A Galli M Determination of quality of life in adolescents with idiopathic scoliosis subjected to conservative treatment. Scoliosis. (2010) 5:21. 10.1186/1748-7161-5-21 20920196 PMC2958155

[60] de Mauroy JC Journe A Gagaliano F Lecante C Barral F Pourret S. The new Lyon ARTbrace versus the historical Lyon brace: a prospective case series of 148 consecutive scoliosis with short time results after 1 year compared with a historical retrospective case series of 100 consecutive scoliosis; SOSORT award 2015 winner. Scoliosis. (2015) 10:26. 10.1186/s13013-015-0047-6 26300954 PMC4545553

[61] Schreiber S Parent EC Moez EK Hedden DM Hill D Moreau MJ The effect of Schroth exercises added to the standard of care on the quality of life and muscle endurance in adolescents with idiopathic scoliosis-an assessor and statistician blinded randomized controlled trial: “SOSORT 2015 award winner”. Scoliosis. (2015) 10:24. 10.1186/s13013-015-0048-5 26413145 PMC4582716

[62] Monticone M Ambrosini E Cazzaniga D Rocca B Ferrante S. Active self-correction and task-oriented exercises reduce spinal deformity and improve quality of life in subjects with mild adolescent idiopathic scoliosis. Results of a randomised controlled trial. Eur Spine J. (2014) 23:1204–14. 10.1007/s00586-014-3241-y 24682356

[63] Rigo MD Villagrasa M Gallo D. A specific scoliosis classification correlating with brace treatment: description and reliability. Scoliosis. (2010) 5:1. 10.1186/1748-7161-5-1 20205842 PMC2825498

[64] Durmala J Dobosiewicz K Jendrzejek H Pius W. Exercise efficiency of girls with idiopathic scoliosis based on the ventilatory anaerobic threshold. Stud Health Technol Inform. (2002) 91:357–60. 10.3233/978-1-60750-935-6-357 15457755

[65] Betts T. The development of a classification system for the treatment of scoliosis by the side shift. Scoliosis. (2014) 9:O66. 10.1186/1748-7161-9-S1-O66

[66] Białek M. Mild angle early onset idiopathic scoliosis children avoid progression under fits method (functional individual therapy of scoliosis). Medicine (Baltimore). (2015) 94:e863. 10.1097/MD.0000000000000863 25997065 PMC4602882

[67] Nisser J Smolenski U Sliwinski GE Schumann P Heinke A Malberg H The fed-method (fixation, elongation, derotation)—a machine-supported treatment approach to patients with idiopathic scoliosis—systematic review. Z Orthop Unfall. (2020) 158:318–32. 10.1055/a-0881-3430 31404938

[68] Janicki JA Poe-Kochert C Armstrong DG Thompson GH. A comparison of the thoracolumbosacral orthoses and providence orthosis in the treatment of adolescent idiopathic scoliosis: results using the new SRS inclusion and assessment criteria for bracing studies. J Pediatr Orthop. (2007) 27:369–74. 10.1097/01.bpb.0000271331.71857.9a 17513954

[69] Ghorbani F Ranjbar H Kamyab M Babaee T Kamali M Razavi H Effect of brace treatment on craniovertebral to lumbopelvic sagittal parameters in adolescents with idiopathic scoliosis: a systematic review. Asian Spine J. (2023) 17:401–17. 10.31616/asj.2022.0011 36625021 PMC10151630

[70] Cheung J Chong C Cheung P. Underarm bracing for adolescent idiopathic scoliosis leads to flatback deformity: the role of sagittal spinopelvic parameters. Bone Joint J. (2019) 101-B:1370–8. 10.1302/0301-620X.101B11.BJJ-2019-0515.R1 31674249

[71] Guo J Lam TP Wong MS Ng BK Lee KM Liu KL A prospective randomized controlled study on the treatment outcome of Spinecor brace versus rigid brace for adolescent idiopathic scoliosis with follow-up according to the SRS standardized criteria. Eur Spine J. (2014) 23:2650–7. 10.1007/s00586-013-3146-1 24378629

[72] Howard A Wright JG Hedden D. A comparative study of TLSO, Charleston, and Milwaukee braces for idiopathic scoliosis. Spine (Phila Pa 1976). (1998) 23:2404–11. 10.1097/00007632-199811150-00009 9836354

[73] Dolan LA Weinstein SL. Surgical rates after observation and bracing for adolescent idiopathic scoliosis: an evidence-based review. Spine (Phila Pa 1976). (2007) 32:S91–100. 10.1097/BRS.0b013e318134ead9 17728687

[74] Moradi V Babaee T Shariat A Khosravi M Saeedi M Parent-Nichols J Predictive factors for outcomes of overcorrection nighttime bracing in adolescent idiopathic scoliosis: a systematic review. Asian Spine J. (2022) 16:598–610. 10.31616/asj.2021.0037 34304236 PMC9441430

[75] Bagnall KM Grivas TB Alos N Asher M Aubin CE Burwell GR The international research society of spinal deformities (IRSSD) and its contribution to science. Scoliosis. (2009) 4:28. 10.1186/1748-7161-4-28 20025783 PMC2808165

[76] Climent JM Sánchez J. Impact of the type of brace on the quality of life of adolescents with spine deformities. Spine (Phila Pa 1976). (1999) 24:1903–8. 10.1097/00007632-199909150-00007 10515014

[77] Babaee T Kamyab M Ganjavian MS Kamali M. Milwaukee brace or thoracolumbosacral orthosis? Which one affects the quality of life of adolescents with idiopathic scoliosis more? A cross-sectional study using the SRS-22 questionnaire. Curr Orthop Pract. (2014) 25:478–83. 10.1097/BCO.0000000000000138

[78] Cheung JPY Cheung PWH Yeng WC Chan LCK. Does curve regression occur during underarm bracing in patients with adolescent idiopathic scoliosis? Clin Orthop Relat Res. (2020) 478:334–45. 10.1097/CORR.0000000000000989 31688210 PMC7438132

[79] de Mauroy JC Lecante C Barral F Pourret S. Prospective study and new concepts based on scoliosis detorsion of the first 225 early in-brace radiological results with the new Lyon brace: ARTbrace. Scoliosis. (2014) 9:19. 10.1186/1748-7161-9-19 25741377 PMC4349706

[80] Aulisa AG Guzzanti V Galli M Perisano C Falciglia F Aulisa L. Treatment of thoraco-lumbar curves in adolescent females affected by idiopathic scoliosis with a progressive action short brace (PASB): assessment of results according to the SRS committee on bracing and nonoperative management standardization criteria. Scoliosis. (2009) 4:21. 10.1186/1748-7161-4-21 19765288 PMC2754424

[81] Katz DE Herring JA Browne RH Kelly DM Birch JG. Brace wear control of curve progression in adolescent idiopathic scoliosis. J Bone Joint Surg Am. (2010) 92:1343–52. 10.2106/JBJS.I.01142 20516309

[82] Havey R Gavin T Patwardhan A Pawelczak S Ibrahim K Andersson GB A reliable and accurate method for measuring orthosis wearing time. Spine (Phila Pa 1976). (2002) 27:211–4. 10.1097/00007632-200201150-00018 11805670

[83] Lou E Hill DL Raso JV. A wireless sensor network system to determine biomechanics of spinal braces during daily living. Med Biol Eng Comput. (2010) 48:235–43. 10.1007/s11517-010-0575-4 20094808

[84] Takemitsu M Bowen JR Rahman T Glutting JJ Scott CB. Compliance monitoring of brace treatment for patients with idiopathic scoliosis. Spine (Phila Pa 1976). (2004) 29:2070–4. 2074. 10.1097/01.brs.0000138280.43663.7b 15371711

[85] Sanders JO Newton PO Browne RH Katz DE Birch JG Herring JA. Bracing for idiopathic scoliosis: how many patients require treatment to prevent one surgery? J Bone Joint Surg Am. (2014) 96:649–53. 10.2106/JBJS.M.00290 24740661

[86] Aubin CE Labelle H Ruszkowski A Petit Y Gignac D Joncas J Variability of strap tension in brace treatment for adolescent idiopathic scoliosis. Spine (Phila Pa 1976). (1999) 24:349–54. 10.1097/00007632-199902150-00010 10065519

[87] Lou E Hill D Hedden D Mahood J Moreau M Raso J. An objective measurement of brace usage for the treatment of adolescent idiopathic scoliosis. Med Eng Phys. (2011) 33:290–4. 10.1016/j.medengphy.2010.10.016 21112234

[88] Xu L Qin X Qiu Y Zhu Z. Initial correction rate can be predictive of the outcome of brace treatment in patients with adolescent idiopathic scoliosis. Clin Spine Surg. (2017) 30:E475–9. 10.1097/BSD.0000000000000343 28437355

[89] van den Bogaart M van Royen BJ Haanstra TM de Kleuver M Faraj S. Predictive factors for brace treatment outcome in adolescent idiopathic scoliosis: a best-evidence synthesis. Eur Spine J. (2019) 28:511–25. 10.1007/s00586-018-05870-6 30607519

[90] Babaee T Kamyab M Ganjavian MS Rouhani N Khorramrouz A Jarvis JG. Coronal deformity angular ratio as a predictive factor for in-brace curve correction and long-term outcome of brace treatment in adolescents with idiopathic scoliosis. Spine Deform. (2022) 10:543–51. 10.1007/s43390-021-00452-x 35034344

[91] Hawary RE Zaaroor-Regev D Floman Y Lonner BS Alkhalife YI Betz RR. Brace treatment in adolescent idiopathic scoliosis: risk factors for failure-a literature review. Spine J. (2019) 19:1917–25. 10.1016/j.spinee.2019.07.008 31325626

[92] Abel MF. Brace success as related to curve type, compliance, and maturity in adolescents with idiopathic scoliosis: commentary on an article by Rachel M. Thompson, MD, et al.: “brace success is related to curve type in patients with adolescent idiopathic scoliosis”. J Bone Joint Surg Am. (2017) 99:e59. 10.2106/JBJS.17.00043 28590389

[93] Sun W Zhou J Sun M Qin X Qiu Y Zhu Z Low body mass index can be predictive of bracing failure in patients with adolescent idiopathic scoliosis: a retrospective study. Eur Spine J. (2017) 26:1665–9. 10.1007/s00586-016-4839-z 27807774

[94] Karol LA Virostek D Felton K Jo C Butler L. The effect of the risser stage on bracing outcome in adolescent idiopathic scoliosis. J Bone Joint Surg Am. (2016) 98:1253–9. 10.2106/JBJS.15.01313 27489315

[95] Karol LA Wingfield JJ Virostek D Felton K Jo C. The influence of body habitus on documented brace wear and progression in adolescents with idiopathic scoliosis. J Pediatr Orthop. (2020) 40:e171–5. 10.1097/BPO.0000000000001420 31259783

[96] Aulisa AG Guzzanti V Marzetti E Giordano M Falciglia F Aulisa L. Brace treatment in juvenile idiopathic scoliosis: a prospective study in accordance with the SRS criteria for bracing studies—SOSORT award 2013 winner. Scoliosis. (2014) 9:3. 10.1186/1748-7161-9-3 24817906 PMC4016774

[97] Shi B Mao S Xu L Sun X Zhu Z Qian B Integrated multidimensional maturity assessments predicting the high-risk occurrence of peak angle velocity during puberty in progressive female idiopathic scoliosis. Clin Spine Surg. (2017) 30:E491–6. 10.1097/BSD.0000000000000203 28437358

[98] Nault ML Parent S Phan P Roy-Beaudry M Labelle H Rivard M. A modified Risser grading system predicts the curve acceleration phase of female adolescent idiopathic scoliosis. J Bone Joint Surg Am. (2010) 92:1073–81. 10.2106/JBJS.H.01759 20439651

[99] Tanner JM Whitehouse RH. Clinical longitudinal standards for height, weight, height velocity, weight velocity, and stages of puberty. Arch Dis Child. (1976) 51:170–9. 10.1136/adc.51.3.170 952550 PMC1545912

[100] Sanders JO Browne RH McConnell SJ Margraf SA Cooney TE Finegold DN. Maturity assessment and curve progression in girls with idiopathic scoliosis. J Bone Joint Surg Am. (2007) 89:64–73. 10.2106/JBJS.F.00067 17200312

[101] Verma K Sitoula P Gabos P Loveland K Sanders J Verma S Simplified skeletal maturity scoring system: learning curve and methods to improve reliability. Spine (Phila Pa 1976). (2014) 39:E1592–8. 10.1097/BRS.0000000000000653 25503941

[102] Hung AL Shi B Chow SK Chau WW Hung VW Wong RM Validation study of the thumb ossification composite index (TOCI) in idiopathic scoliosis: a stage-to-stage correlation with classic tanner-whitehouse and sanders simplified skeletal maturity systems. J Bone Joint Surg Am. (2018) 100:88. 10.2106/JBJS.17.01271 29975274 PMC6075884

[103] Lau L Hung A Chau WW Hu Z Kumar A Lam TP Sequential spine-hand radiography for assessing skeletal maturity with low radiation EOS imaging system for bracing treatment recommendation in adolescent idiopathic scoliosis: a feasibility and validity study. J Child Orthop. (2019) 13:385–92. 10.1302/1863-2548.13.190007 31489044 PMC6701449

[104] Luk KD Saw LB Grozman S Cheung KM Samartzis D. Assessment of skeletal maturity in scoliosis patients to determine clinical management: a new classification scheme using distal radius and ulna radiographs. Spine J. (2014) 14:315–25. 10.1016/j.spinee.2013.10.045 24239801

[105] Hans SD Sanders JO Cooperman DR. Using the sauvegrain method to predict peak height velocity in boys and girls. J Pediatr Orthop. (2008) 28:836–9. 10.1097/BPO.0b013e31818ee3c4 19034174

[106] Sauvegrain J Nahum H Bronstein H. Study of bone maturation of the elbow. Ann Radiol (Paris). (1962) 5:542–50. 13986863

[107] Abbassi V. Growth and normal puberty. Pediatrics. (1998) 102:507–11. 10.1542/peds.102.S3.507 9685454

[108] Mao SH Jiang J Sun X Zhao Q Qian BP Liu Z Timing of menarche in Chinese girls with and without adolescent idiopathic scoliosis: current results and review of the literature. Eur Spine J. (2011) 20:260–5. 10.1007/s00586-010-1649-6 21153847 PMC3030718

[109] Sanders JO. Maturity indicators in spinal deformity. J Bone Joint Surg Am. (2007) 89(Suppl 1):14–20. 10.2106/JBJS.F.00318 17272419

